# Mixotrophy as an alternative strategy under iron-limited upwelling conditions

**DOI:** 10.1093/ismeco/ycag290

**Published:** 2026-10-08

**Authors:** Emily V Speciale, Prisca Lim, Yubeen Jeong, Claire C Z Cook, Natalie R Cohen, Will McClure, Astrid Schnetzer, Claire P Till, Ralph C Till, Adrian Marchetti

**Affiliations:** Department of Earth, Marine, and Environmental Sciences, University of North Carolina at Chapel Hill, Chapel Hill, NC 27599, United States; Department of Earth, Marine, and Environmental Sciences, University of North Carolina at Chapel Hill, Chapel Hill, NC 27599, United States; Department of Earth, Marine, and Environmental Sciences, University of North Carolina at Chapel Hill, Chapel Hill, NC 27599, United States; Skidaway Institute of Oceanography, University of Georgia, Savannah, GA 31411, United States; Skidaway Institute of Oceanography, University of Georgia, Savannah, GA 31411, United States; Marine, Earth, and Atmospheric Sciences, North Carolina State University, Raleigh, NC 27695, United States; Marine, Earth, and Atmospheric Sciences, North Carolina State University, Raleigh, NC 27695, United States; Chemistry Department, California State Polytechnic University, Humboldt, Arcata, CA 95521, United States; Chemistry Department, California State Polytechnic University, Humboldt, Arcata, CA 95521, United States; Department of Earth, Marine, and Environmental Sciences, University of North Carolina at Chapel Hill, Chapel Hill, NC 27599, United States

**Keywords:** mixotrophy, iron, upwelling, metatranscriptomics, diatoms

## Abstract

Mixotrophs, phytoplankton capable of both phototrophy and heterotrophy, serve crucial yet complex roles in ocean food webs and carbon cycling. As climate change is expected to reduce iron bioavailability within upwelling zones, mixotrophs may have an advantage due to an array of possible iron acquisition strategies. We paired physiological measurements with metatranscriptomics to investigate the molecular mechanisms of mixotrophs within an upwelling zone as a function of iron status. Our study occurred during a biologically productive upwelling season within the California Current System, typically dominated by large phototrophic diatoms. Subsurface waters from an active upwelling site were incubated under control (Ctrl), iron-addition (+Fe), and desferrioxamine B (DFB, a strong iron chelator) treatments. Results revealed differing phytoplankton communities; Ctrl/+Fe communities had high biomass and RNA transcripts dominated by diatoms, whereas the DFB community displayed symptoms of iron limitation, lower biomass, and a higher proportion of putative mixotrophs. Iron-limited mixotrophs reduced expression of photosynthetic processes while highly expressing those linked to iron stress, signal transduction, and the phagolysosome compared to iron-replete mixotrophs. This response contrasted with diatoms, which displayed less change in photosynthetic machinery or signal transduction under iron limitation. Our results suggest mixotrophy as an alternative strategy used by some phytoplankton to cope with iron limitation, and although diatoms dominate under high-iron scenarios, low-iron scenarios may pose a different outcome.

## Introduction

Coastal upwelling regions are areas of high biological productivity that support biogeochemical cycling, efficient food chains, and commercial fisheries [1–3]. One such upwelling region is the California Current System (CCS), in which summertime coastal upwelling gives rise to increased productivity dominated by large, phototrophic diatoms [4–6]. Several models agree that by the end of the century, CCS upwelling patterns will change; northern CCS upwelling will likely intensify, while central and/or southern CCS upwelling will likely weaken [7–9]. These models also unanimously predict increased sea surface temperature (SST) within the CCS, indicating future stratified conditions [7–9].

Changes in upwelling velocities and SST will inevitably impact nutrient fluxes that support this region’s productivity, such as iron. Iron is a vital micronutrient used by phytoplankton for oxygenic photosynthesis [10, 11] and nitrogen assimilation [12], yet is typically limited within the open ocean and parts of the CCS [4, 13, 14].Iron bioavailability could become even more limited under both strengthened and weakened upwelling conditions. Strengthened upwelling would increase CO_2_ flux to surface waters, contributing to ocean acidification, which recent studies have shown could reduce iron bioavailability and increase iron stress in phytoplankton [15, 16]. Alternatively, weakened upwelling caused by stratification would reduce water column mixing, thus reducing nutrient flux (including iron) to surface waters with impacts on phytoplankton productivity, as seen in the Iberian coastal upwelling system [17]. Diatoms in the CCS will likely be most affected by changes in iron bioavailability, as their growth and biomass decline significantly under iron stress [4, 18].

While some diatoms may be disadvantaged within iron-limited environments, other phytoplankton groups may have an advantage. One such group is mixotrophic phytoplankton, or mixoplankton [19], which includes constitutive “mixotrophs” that retain the ancestral ability to consume prey via phagotrophy [20]. Phagotrophy, or phagocytosis, is the engulfment and digestion of large particles including organisms such as bacteria or small phytoplankton [20]. Some mixotrophs have the ability to obtain iron directly through the phagotrophic ingestion of bacteria or colloids [21, 22]. Mixotrophs may have higher abundance in oligotrophic waters where their two modes of nutritional uptake can provide an advantage [23, 24], and the usage of mixotrophy could result in higher biomass retention that increases food web and biological carbon pump efficiency within an ecosystem [25–27]. Alternatively, several mixotrophic species are responsible for harmful algal blooms, which can disrupt and alter food web dynamics [28, 29]. Given the likelihood of future iron limitation within the CCS, it is important to understand how mixotrophs will adjust to iron-limited upwelling conditions, yet these mechanisms remain largely uncharacterized.

Examining gene expression via RNA-seq is a common technique used to understand how phytoplankton adapt under variable environments. Several transcriptomic studies were conducted on mixotrophic species under varying conditions such as light, prey, and macronutrient availability [30–34]. These studies describe baseline genes that change in expression when a phytoplankton enacts its mixotrophic capabilities. However, these transcriptomic studies only focus on single species and were conducted under controlled laboratory conditions. In this study, metatranscriptomics was used to investigate mixotrophy under variable iron conditions. Metatranscriptomic studies have already discovered how some natural phytoplankton communities, particularly those dominated by diatoms, respond to iron stress [18, 35] but are scarce for mixotrophs [36, 37].

We combined physiological measurements with metatranscriptomics to investigate the effect of iron status on mixotroph gene expression under upwelling conditions. First, we present physiological results that confirm low iron bioavailability as an inhibitor on the growth and productivity of the phytoplankton community. Next, we show the impact of iron status on taxonomic transcriptional activity, as iron-limited conditions result in higher proportions of mixotrophs. Finally, we analyze the gene expression patterns of mixotrophs under iron-replete and iron-limited conditions, and we compare this gene response to that of phototrophic diatoms.

## Materials and Methods

Data collection took place on the R/V Sally Ride from 29 May to 10 June 2023. To simulate upwelling conditions under different iron treatments, an onboard incubation experiment was conducted. Seawater for the experiment was collected within the northern CCS, off the southern coast of Oregon at 43°02′42.7”N, 124°33′07.2”W. The collected seawater was deemed as freshly upwelled water and taken from a depth of 55 m—receiving <1% irradiance—using trace-metal clean techniques on 30 May 2023, 13:30 Greenwich Mean Time (GMT). The sampled depth was selected to perform the experiment with a subsurface phytoplankton population from just below the euphotic zone that could then be experimentally brought into the light to simulate upwelling under the varying iron treatments. The seawater was pumped and homogenized using trace metal clean techniques, then transferred into a total of 30 20 L low-density polyethylene cubitainers. Three cubitainers were immediately harvested for the initial timepoint (T0). The remaining 27 cubitainers were assigned treatments: 9 were unamended (Ctrl), 9 were amended with 5 nM FeCl_2_ (+Fe), and 9 were amended with 200 nM desferrioxamine B, a strong iron chelator that inhibits dissolved iron uptake (DFB). The cubitainers were placed in an on-deck incubator covered with two layers of neutral density screening to simulate 26% of incidence irradiance supplied with flow-through surface seawater to maintain ambient surface temperature. Three cubitainers from each treatment were harvested for each of the three subsequent timepoints: 48 h (T1), 168 h (T2), and 264 h (T3) after incubation. Seawater from the cubitainers was then partitioned and distributed appropriately to perform all measurements described below. While T1 was aimed at capturing the early response to the treatments, T2 and T3 were aimed at capturing late exponential phase and early stationary phase growth, respectively. These incubation time scales are broadly consistent with physical residence and transport time scales in coastal upwelling systems, especially in the CCS [38, 39].

### Physiological data collection

Dissolved inorganic nutrient measurements were analyzed for nitrate (NO_3_^−^), phosphate (PO_4_^3−^), silicic acid [Si(OH)_4_], and iron (dFe). Size-fractionated isotope uptake rates of dissolved inorganic carbon (DIC) and NO_3_^−^ were assessed using 6-h incubations; absolute uptake rates (ρ) and biomass-normalized uptake rates (*V*) of DIC and NO_3_^−^ were calculated using the constant transport model and the specific uptake model, respectively [40].

Size-fractionated chlorophyll *a* (chl *a*) measurements were analyzed for large (≥5 μm) and small (<5 μm) cell concentrations. The 5 μm size-fractionation threshold was selected to distinguish diatoms and other large phytoplankton (e.g. dinoflagellates) from smaller phytoplankton groups such as cyanobacteria, picoeukaryotes, and nanoflagellates. While diatoms in our study region are largely confined to the large size-fraction, mixotrophs span both size-fractions [41]. Concentrations of nanoeukaryotes, picoeukaryotes, *Synechococcus*, and bacteria were also assessed using flow cytometry [42]. FlowCAM analyses of broad diatom taxa groups were performed for replicates within T2 [43]. Additionally, 1-h fluorescently labeled particle (FLP) incubations were conducted with 0.5 μm green microspheres and analyzed via epifluorescent microscopy to determine cell-specific grazing rates (CSGRs) of mixotrophs within each sample [42].

Analysis of Variance (ANOVA) and Tukey tests were conducted to determine significance between treatments at each timepoint (α = 0.05), and all ANOVA *P*-values are provided in Table S1. Detailed protocols for physiological measurements are provided in the Supplementary Text.

### RNA collection and metatranscriptomics

Cubitainers were harvested for RNA by collecting and filtering ~2.5–4 L of each sample onto 0.8 μm Pall Supor filters (142 mm) using a peristaltic pump; samples were then immediately flash-frozen in liquid nitrogen and stored at −80°C. RNA was extracted using the RNAqueous-4PCR kit, per instructions from the manufacturer with few modifications—filters were cut up, 200 μl of glass beads were added, and 3 ml of lysis buffer was added. RNA samples were sent to GENEWIZ for library preparation and sequencing with PolyA tail selection. Sequencing was performed on an Illumina HiSeq 4000 with a 2 × 150 bp configuration. GENEWIZ provided raw paired-end read sequences for each sample.

Raw reads were trimmed using Trim Galore v0.6.10, and quality control was assessed through FastQC [44]. A *de novo* metatranscriptome assembly was conducted using rnaSPAdes v3.15.5 for individual assemblies [45] and CD-HIT v4.8.1 for grand assembly [46]. TransDecoder v5.7.1 was used to determine the most likely open reading frame (ORF) for each contig [47]. ORFs were annotated using the Marine Functional Eukaryotic Reference Taxa (MarFERReT) database v1.1.1 (*e*-value <1*e*^−06^) [48] for National Center for Biotechnology Information (NCBI) taxonomies [49], Protist Ribosomal Reference (PR^2^) taxonomies [50], and Pfam 34.0 functions [51]. To remove any potential bacterial contamination, ORFs were also annotated against the PhyloDB v1076 database using EUKulele (*e*-value <1*e*^−06^) [52]. Any ORF with a higher bitscore for a bacteria/virus compared to its MarFERReT annotation was removed (resulting in <3% of ORFs removed). To maximize functional coverage, ORFs were annotated using eggNOG-mapper v2.1.12 [53] to obtain annotations from the Kyoto Encyclopedia of Genes and Genomes (KEGG) [54]. Trimmed samples were then aligned to the grand assembly using Salmon v1.10.3 [55] and exported into a counts table using tximport [56]. Reads not mapping to a protist group as defined by MarFERReT annotations were removed before downstream analysis.

DESeq2 was used to obtain normalized counts and conduct differential expression analyses [57]. For taxonomic composition, all protist reads were normalized using DESeq2’s median of ratios methods and then presented on a proportional scale. Differential expression analyses (for Pfam protein domains, KEGG genes, and KEGG pathways) were performed for reads mapping to mixotroph genera and reads mapping to the lineage Bacillariophyta (henceforth referred to as diatoms). Reads not mapping to the taxa group being analyzed were removed prior to normalization and statistical testing. Reads were summed based on annotation ID prior to entering DESeq2 analysis, as done in previous metatranscriptomic iron studies in the CCS [18, 58]; if a contig annotated to multiple IDs, its reads were evenly distributed between the mapped annotations.

Previous metatranscriptomic studies have used machine learning to predict *in situ* trophic modes of protist species [36, 37]. We chose to identify mixotrophs based on constitutive mixotroph genera as identified within the Mixoplankton Database (MDB) [41]. We chose to group by genera because the resolution of annotation provided by metatranscriptomics may not be reliable enough to define transcripts on a species level [59]. Ultimately, using this database provided a more streamlined approach in grouping together as many potential mixotrophs as possible. Although mixotrophs are heterogenous, with each genera/species having its own trophic mode preferences and unique responses to the environmental conditions, our approach allows for understanding of the broad transcriptional responses of this diverse trophic group and thus may be confounded by both changes in physiology and mixotrophic community composition.

## Results

An iron incubation experiment was conducted in June 2023 in which natural subsurface phytoplankton communities in an active northern CCS upwelling site (Fig. S1) were collected and brought to sunlight within on-deck incubators to simulate upwelling conditions. Three treatments were assigned: no amendments (Ctrl), an iron-addition amendment using 5 nM FeCl_2_ (+Fe), and an iron-limiting amendment using 200 nM desferrioxamine B (DFB) (Fig. S1). Data was collected 0 h (T0), 48 h (T1), 168 h (T2), and 264 h (T3) after incubation. While microbial subsurface upwelling communities vary spatiotemporally within the CCS, we believe our experiment provides valuable insights on the dynamics of mixotrophs in response to variable iron conditions.

### Inorganic dissolved nutrients and uptake rates

Macronutrient concentrations of nitrate (NO_3_^−^) and silicic acid [Si(OH)_4_] differed most drastically between treatments at T2, where appreciable drawdowns occurred in Ctrl and + Fe (Fig. 1A and B). This corresponded with a spike in biomass-normalized uptake rates of dissolved inorganic carbon (*V*DIC) for large cells (≥5 μm) at T2 (Fig. 1E). NO_3_^−^ was almost completely depleted in Ctrl and + Fe at T2, resulting in low values of *V*NO_3_^−^ in Ctrl and + Fe for both large and small (<5 μm) size-fractions (Fig. 1G and H). In contrast, all macronutrients in DFB displayed minimal drawdown by T2, resulting in significant differences between DFB and Ctrl/+Fe (*P* < .001). Large-cell *V*DIC was significantly lower in DFB compared to Ctrl/+Fe (*P* = 6e^−04^), while small-cell *V*NO_3_^−^ was significantly higher in DFB at T2. At T3, NO_3_^−^ was completely depleted in Ctrl and + Fe, whereas NO_3_^−^ in DFB decreased, resulting in further significant differences between treatments (*P* = 2e^−04^). Si(OH)_4_ concentrations, which can indicate diatom growth, showed similar patterns to NO_3_^−^; Si(OH)_4_ significantly declined in Ctrl and + Fe by T2 (*P* = .006), whereas in DFB, concentrations declined after T2. Phosphate (PO_4_^3−^) showed very similar trends to NO_3_^−^ and Si(OH)_4_ (Table S1) but was not visualized due to redundancy. Absolute uptake rates (ρ) of DIC and NO_3_^−^ showed similar trends to *V*DIC and *V*NO_3_^−^ and can be found in Fig. S2.

**Figure 1:**
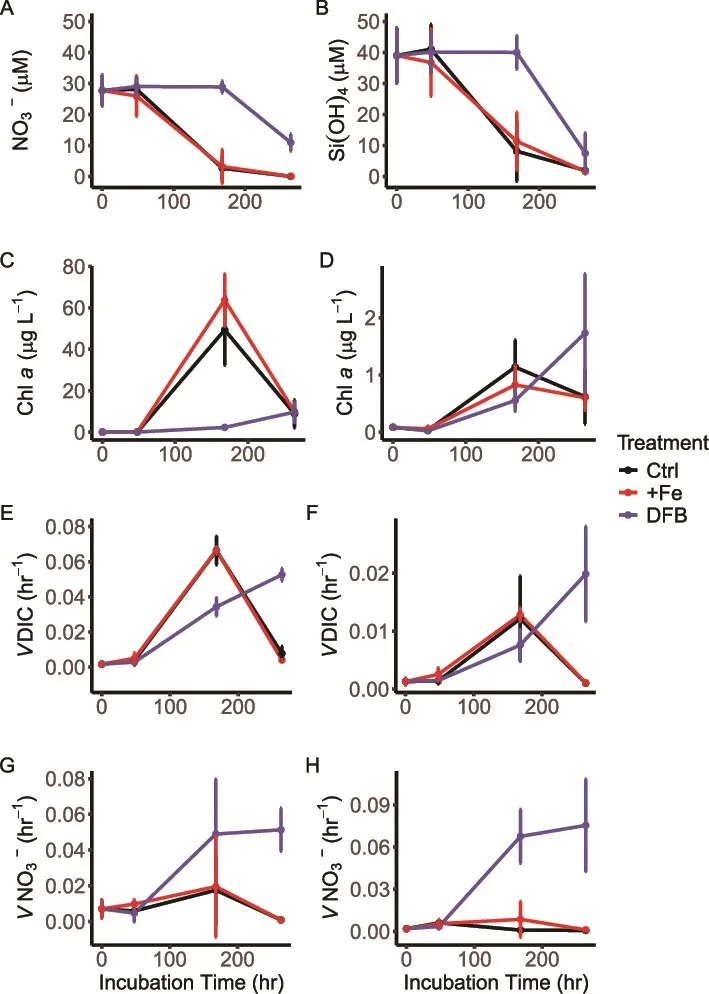
Physiological measurements (mean ± standard deviation) during the iron incubation experiment. Control (Ctrl), iron-addition (+Fe), and desferrioxamine B addition (DFB) treatments are colored black, red, and blue respectively. Data points correspond to 0 h (T0), 48 h (T1), 168 h (T2), and 264 h (T3) after incubation. (A) Dissolved inorganic concentrations (μmol L^−1^, or μM) of nitrate (NO_3_^−^) and (B) silicic acid [Si(OH)_4_]. (C) Size-fractionated chlorophyll *a* (chl *a*, μg L^−1^) for large cells (≥5 μm) and (D) small cells (<5 μm). (E) Biomass normalized uptake rates of dissolved inorganic carbon (*V*DIC, h^−1^) for large cells and (F) small cells. (G) Biomass normalized uptake rates of nitrate (*V*NO_3_^−^, h^−1^) for large cells and (H) small cells.

Dissolved iron concentrations (dFe) were analyzed and can be found in Table S2. Initial concentrations were 6.11 ± 0.12 nmol/kg, implying high iron bioavailability at the upwelling site. dFe was similar between Ctrl and + Fe at all timepoints, with a substantial decrease from T1 to T2. dFe in DFB was high across timepoints (>13 nmol/kg); however, this was because samples were UV-irradiated to release iron from DFB prior to analysis. Thus, most of the dFe measured was likely not bioavailable and instead was initially bound to DFB, and the high values indicate the strength of DFB as an iron chelator within the experiment.

### Phytoplankton biomass

Size-fractionated chlorophyll *a* (chl *a*) concentrations aligned with nutrient patterns (Fig. 1C and D). Chl *a* remained relatively low in all treatments at T1, but for large cells, chl *a* in DFB was significantly lower than Ctrl/+Fe (*P* = .004). Large cell chl *a* at T2 was significantly higher in Ctrl/+Fe compared to DFB (*P* = .002). Small cell chl *a* increased in all treatments at T2 but was not significantly different between treatments. At T3, chl *a* for both size fractions decreased in Ctrl and + Fe but increased in DFB, resulting in no significant differences. Biomass differences between treatments were further validated by nanoeukaryote concentrations via flow cytometry, in which similar trends were present (Fig. S3).

### Taxonomic composition

Across 30 samples, a total of 797 714 869 RNA reads were produced, averaging 26 million ± 7 million reads per sample (Table S3). Prior to downstream analysis, any non-protist reads were removed, resulting in nine taxonomic lineage groups [48]. The remaining reads were analyzed using DESeq2 normalization [57] for their taxonomic makeup relative to all protist reads regarding the whole community (Fig. 2A), potential mixotrophs (Fig. 2B), and diatoms (Fig. 2C). Relative abundance results within mixotrophs and diatoms are also shown in Fig. S4.

**Figure 2:**
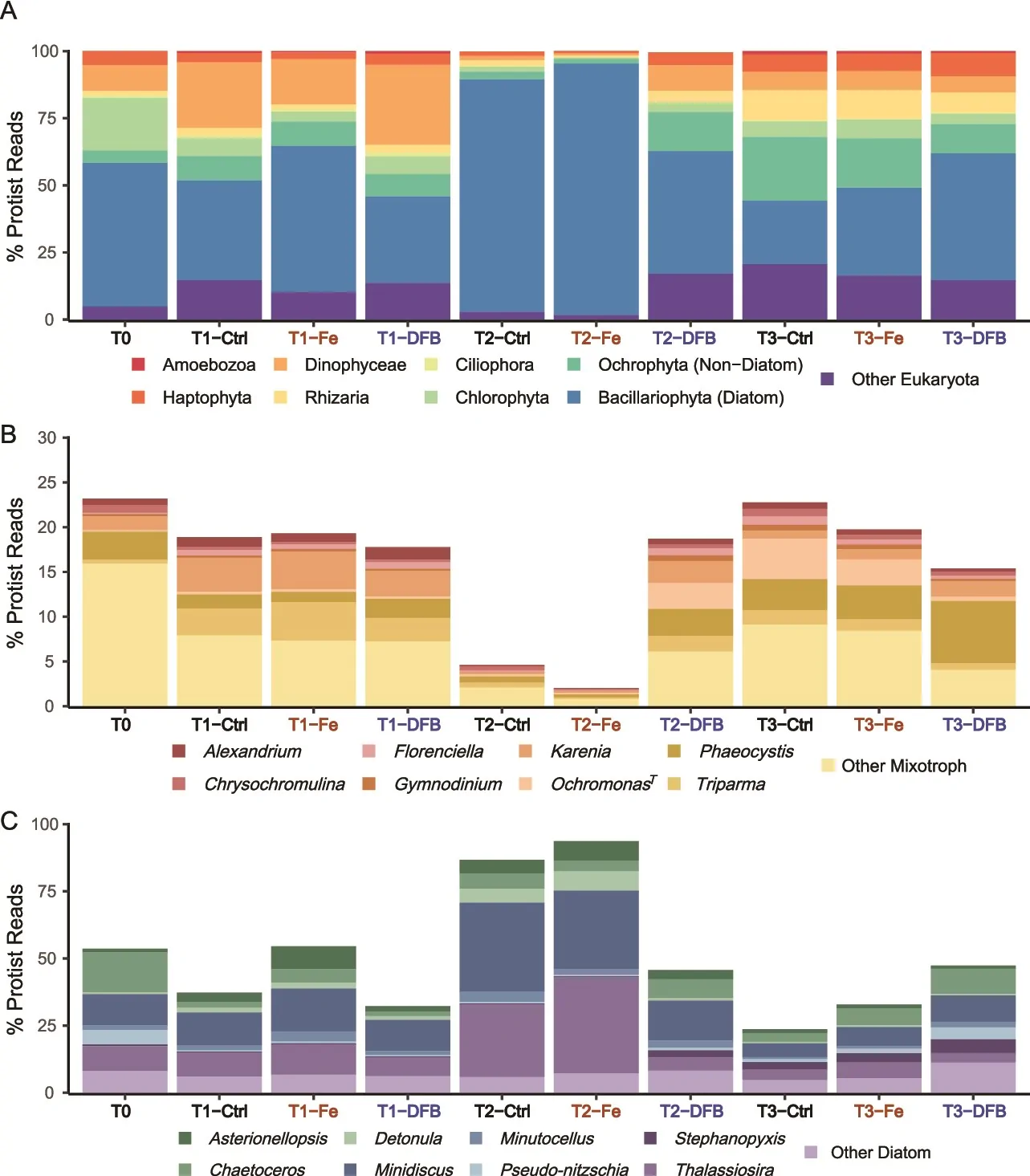
Mean proportional taxonomic makeup of RNA read counts for each timepoint/treatment; any read not mapping to a protist lineage was discarded before analysis, then the remaining reads were normalized using DESeq2. Timepoints are 0 h (T0), 48 h (T1), 168 h (T2), and 264 h (T3) after incubation. Treatments are control (Ctrl), iron-addition (+Fe), and desferrioxamine B addition (DFB). (A) Proportion of reads mapping to nine different taxonomic lineages within the protist community. Taxonomic lineages were defined using MarFERReT guidelines. (B) Proportion of reads mapping to a constitutive mixotroph genus as defined by the Mixoplankton Database; the eight most abundant mixotroph genera are highlighted, with the rest of the genera grouped into “Other Mixotroph.” (C) Proportion of reads mapping to a diatom genus; the eight most abundant diatom genera are highlighted, with the rest of the genera grouped into “Other Diatom.”

At T0, 54.65 ± 4.81% of protist reads mapped to diatoms. The proportion of diatom reads decreased in Ctrl and DFB at T1, whereas the proportion was significantly higher in +Fe (*P* = .025). At T2, diatom reads significantly dominated over three-quarters of the community in Ctrl and + Fe but were less than half of reads in DFB (*P* = 1e^−04^). FlowCAM imaging also supported significantly higher cell counts of diatom genera in Ctrl/+Fe relative to DFB at T2, specifically for *Asterionellopsis* spp*.* (*P* = .014) and *Chaetoceros* spp*. (P* = .037*)* (Fig. S5). In DFB, taxonomy was more diverse, with notable proportions of reads mapping to Ochrophyta, Dinophyceae, Haptophyta, and Other Eukaryota lineages. At T3, Ctrl/+Fe diatom proportions decreased while proportions slightly increased in DFB.

Mixotrophs were identified using constitutive mixotroph genera from the MDB [41]. 35 genera were identified and counted toward mixotroph reads (Table S4). The initial proportion of mixotroph reads was 23.20 ± 3.56%. At T1, the proportion of mixotroph reads decreased but was similar among treatments, ranging from ~17% to 22%. Coinciding with the diatom spike, the proportion of mixotroph reads at T2 significantly declined in Ctrl (4.60 ± 2.88%) and +Fe (2.03 ± 0.29%) but was maintained in DFB (18.71 ± 2.29%) (*P* = 2e^−04^). At T3, the proportion of mixotroph reads increased in Ctrl and +Fe but was variable in DFB.

FLP (i.e. grazing) experiments were also conducted as another relative measure of mixotrophic activity. Results showed no significance between treatments at T1 and T2 (due to variability among replicates), but overall, CSGRs (bacteria mixotroph^−1^ h^−1^) were higher in DFB versus +Fe (Fig. S6). Interestingly, the CSGR was significantly higher in Ctrl/+Fe versus DFB at T3 (*P* = 1.4e^−02^), which coincides with a significant spike in bacterial concentrations at T3 within those treatments (*P* = 3.0e^−03^) (Fig. S3).

### Gene expression trends in mixotrophs and diatoms

Differential expression analyses for protein domains (Pfam), genes (KEGG), and pathways (KEGG) were performed on mixotroph reads [51, 54]. Analyses focused on +Fe vs. DFB treatments from T0 to T2 to compare an iron-replete versus iron-limited environment; Ctrl and T3 were excluded due to large variability among biological replicates, possibly caused by low read and annotation mappings (Table S3). The same analyses were conducted on diatom reads to compare trends of mixotrophs to those of strict phototrophs. A protein domain/gene/pathway was considered differentially expressed if the absolute value of log2FoldChange (L2FC) > 1 and the *P*-adjusted value (*P*-adj) < .05.

For both mixotrophs and diatoms, the number of domains, genes, and pathways differentially expressed between treatments was low at T1 but notably increased at T2 (Table S5). Particularly, differential expression was confined mostly in DFB compared to the other treatments at T1 but higher in +Fe at T2.

#### Nutrient stress

DFB mixotrophs generally increased expression of genes related to iron stress. At T1, DFB mixotrophs highly expressed the low iron-inducible periplasmic protein domain (*Fea1*), a high-affinity iron assimilator secreted under iron deficiency [60, 61] (Fig. 3A). The iron permease FTR1 domain (*FTR1*) is part of the high-affinity iron uptake pathway [61], and DFB mixotrophs had higher expression of the domain at T1 and T2 (Fig. 3A and B). The ferredoxin gene (*petF*), which transcribes an iron-based electron transporter, was highly expressed in +Fe mixotrophs at T2, indicating that this community still had easy access to dissolved iron (Fig. 4). DFB diatoms also highly expressed *Fea1* and *FTR1* at T1/T2 and *Flavodoxin_1* at T1 (Fig. 3C and D). Diatoms in +Fe did not highly express *petF* relative to DFB, implying that iron status did not impact expression of this gene in strict phototrophs.

**Figure 3:**
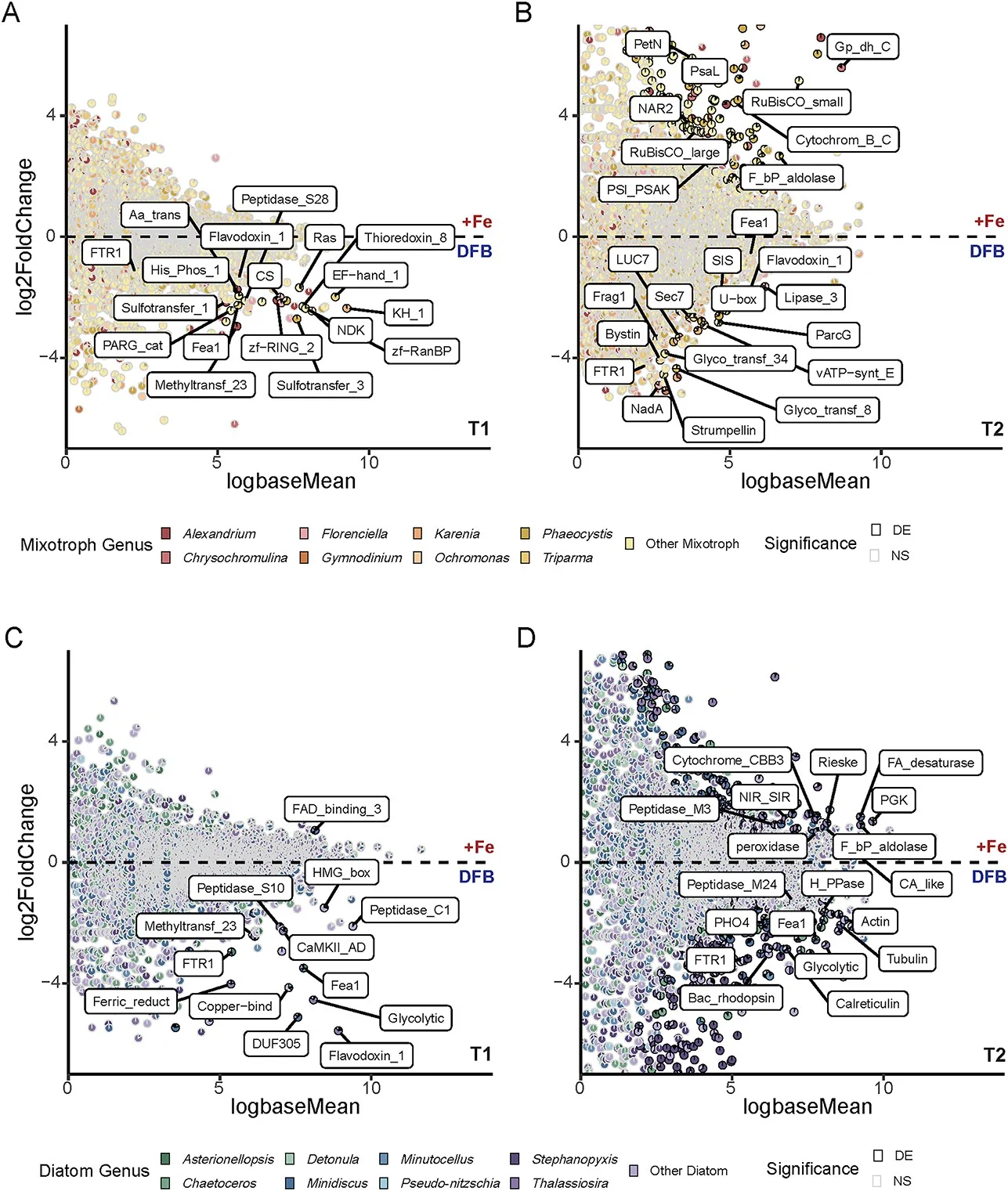
Expression of Pfam domains under iron-addition (+Fe) and desferrioxamine B addition (DFB) conditions for (A) mixotrophs at 48 h (T1), (B) mixotrophs at 168 h (T2), (C) diatoms at T1, and (D) diatoms at T2. The *x*-axis shows the logbaseMean (natural log of the average normalized reads across all samples) and the *y*-axis shows the log2FoldChange (L2FC), where positive values indicate higher expression in +Fe and negative values indicate higher expression in DFB. Each pie chart corresponds to a specific Pfam domain, with segments showing the proportional representation of different mixotroph genera mapping to that domain for the specified timepoint and treatments. Black borders indicate differentially expressed (DE) domains (|L2FC| > 1 & *P*-adj < .05), while grey borders indicate non-significant (NS) domains. Pfam domains of interest are labelled with text, and their corresponding ID, long name, and descriptions can be found in Table S7.

**Figure 4:**
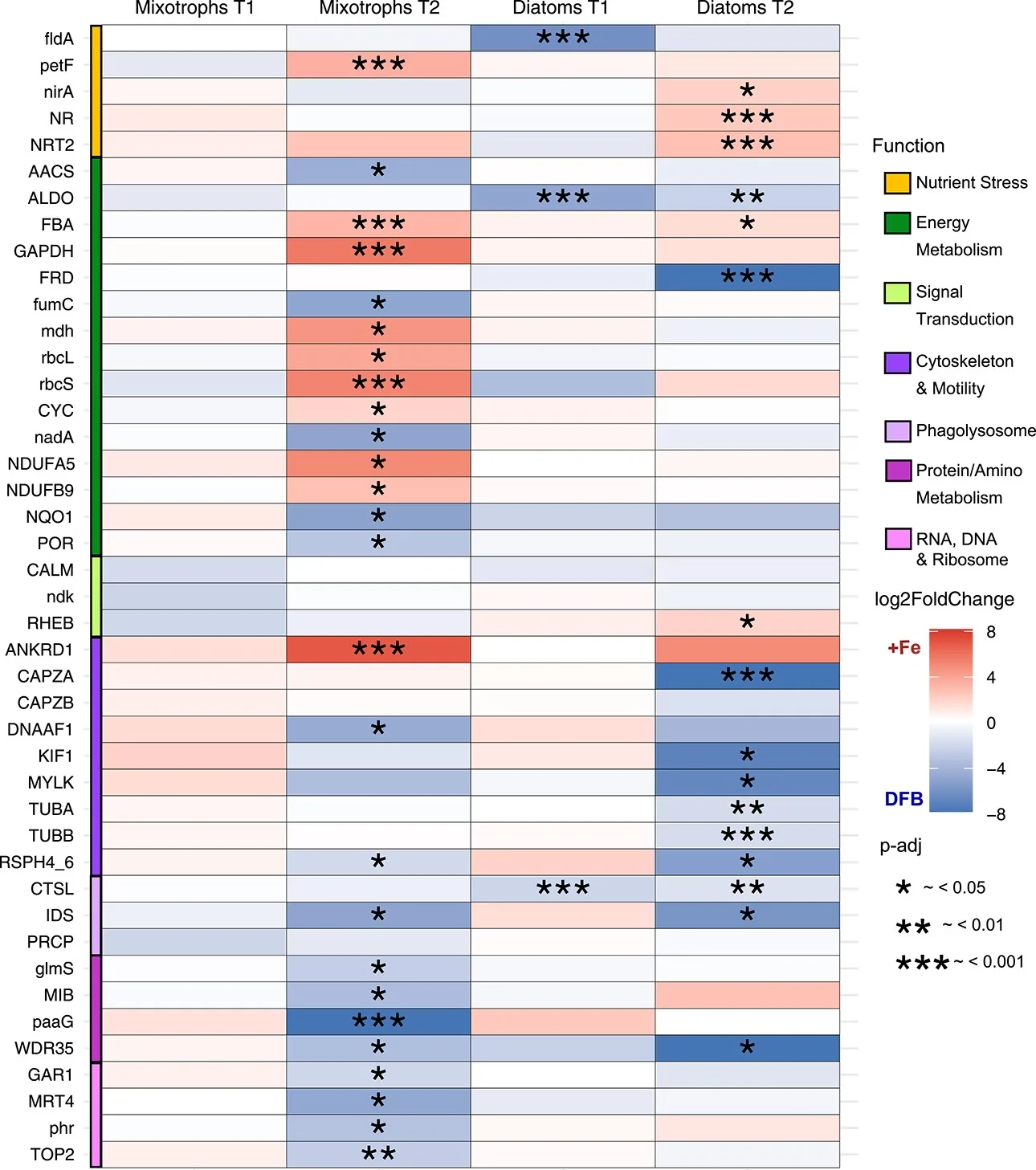
Heatmap of KEGG gene expression within mixotrophs and diatoms at T1 (48 h) and T2 (168 h). Genes are categorized with a border color based on their functional category. Warmer/red colors indicate a positive log2FoldChange (L2FC) and thus higher expression in iron-addition (+Fe) treatment; cooler/blue colors indicate a negative L2FC and thus higher expression in the desferrioxamine B addition (DFB) treatment; and white colors indicate no change in expression between treatments. The *P*-adjusted value (*P*-adj) of differentially expressed genes are represented by stars: one star indicates *P*-adj < .05, two stars indicate *P*-adj < .01, and three stars indicate *P*-adj < .001. The KEGG genes within the heatmap and their corresponding KO IDs and descriptions can be found in Table S8.

Phytoplankton in +Fe were likely beginning to feel the effects of NO_3_^−^ depletion by T2. Mixotrophs in +Fe had increased expression of a high-affinity nitrate transporter domain (*NAR2*) relative to DFB (Fig. 3B), whereas diatoms in +Fe also had higher expression of a ferredoxin-nitrite reductase (*nirA*), nitrate reductase (*NR*), and nitrate/nitrite transporter (*NRT2*) at T2, thus exhibiting a more pronounced response to NO_3_^−^ depletion (Fig. 4).

#### Energy metabolism

Mixotrophs showed stark differences in photosynthetic gene expression between treatments at T2. Mixotrophs in +Fe had significantly higher expression of six photosystem I (PSI) subunits, three photosystem II (PSII) subunits, four cytochrome b6-f complex subunits, three F-type ATPase subunits, and six light-harvesting complexes (LHCs) compared to DFB (Fig. 5). The Calvin cycle (ko00710) and glycolysis/gluconeogenesis pathways (ko00010) were highly expressed in +Fe mixotrophs at T2 (Fig. 6A), as well as genes encoding ribulose bisphosphate carboxylase (*rbcL*, *rbcS*), glyceraldehyde 3-phosphate dehydrogenase (*GAPDH*), fructose-bisphosphate aldolase (*FBA*), and malate dehydrogenase (*mdh*) (Fig. 4). The relatively lower expression of photosynthetic genes in DFB mixotrophs is consistent with prior studies [30, 62]. Regarding carbohydrate metabolism, CAZy enzymes such as glycosyl hydrolases/transferases were highly expressed within grazing protists in numerous studies [31, 32, 63–65]. Within our study, one glycoside hydrolase domain (*Glyco_hydro_5_C*) and two glycosyl transferase domains (*Glyco_transf_8*, *Glyco_transf_34*) were highly expressed in DFB mixotrophs at T2 relative to +Fe (Fig. 3B). Glycerolipid metabolism (ko00561) and glycerophospholipid metabolism (ko00564) pathways were also highly expressed in DFB mixotrophs at T1 (Fig. 6A), along with the Lipase (class 3) protein domain (*Lipase_3*) at T2 (Fig. 3B).

**Figure 5:**
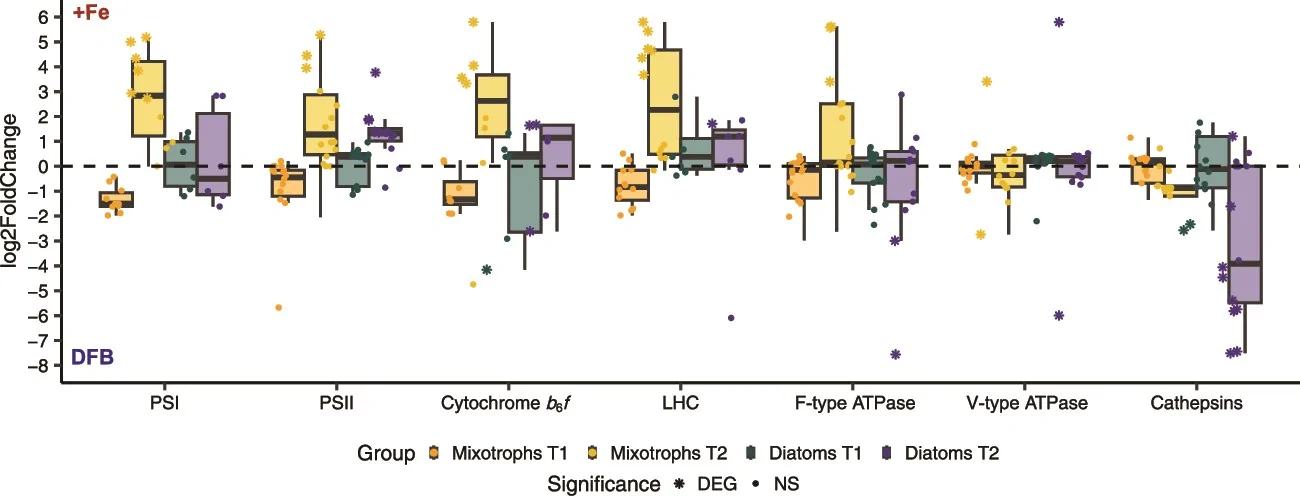
Box plots of KEGG gene groups within mixotrophs and diatoms at T1 (48 h) and T2 (168 h). The gene groups include photosystem I (PSI), photosystem II (PSII), cytochrome *b*_6_*f* complexes, light-harvesting complexes (LHCs), F-type ATPases, V-type ATPases, and cathepsins. Names of specific genes plotted for each gene group can be found in Table S9. The *y*-axis shows log2FoldChange (L2FC), where positive values indicate higher expression in iron-addition (+Fe) treatment and negative values indicate higher expression in the desferrioxamine B (DFB) treatment. Mixotrophs are represented by warm colors (orange for T1, gold for T2), while diatoms are shown in cool colors (green for T1, purple for T2). Each point represents the L2FC of an individual gene, with stars indicating differentially expressed genes (DEGs) (|L2FC| > 1, *P*-adj < .05), and dots representing non-significant (NS) genes.

**Figure 6:**
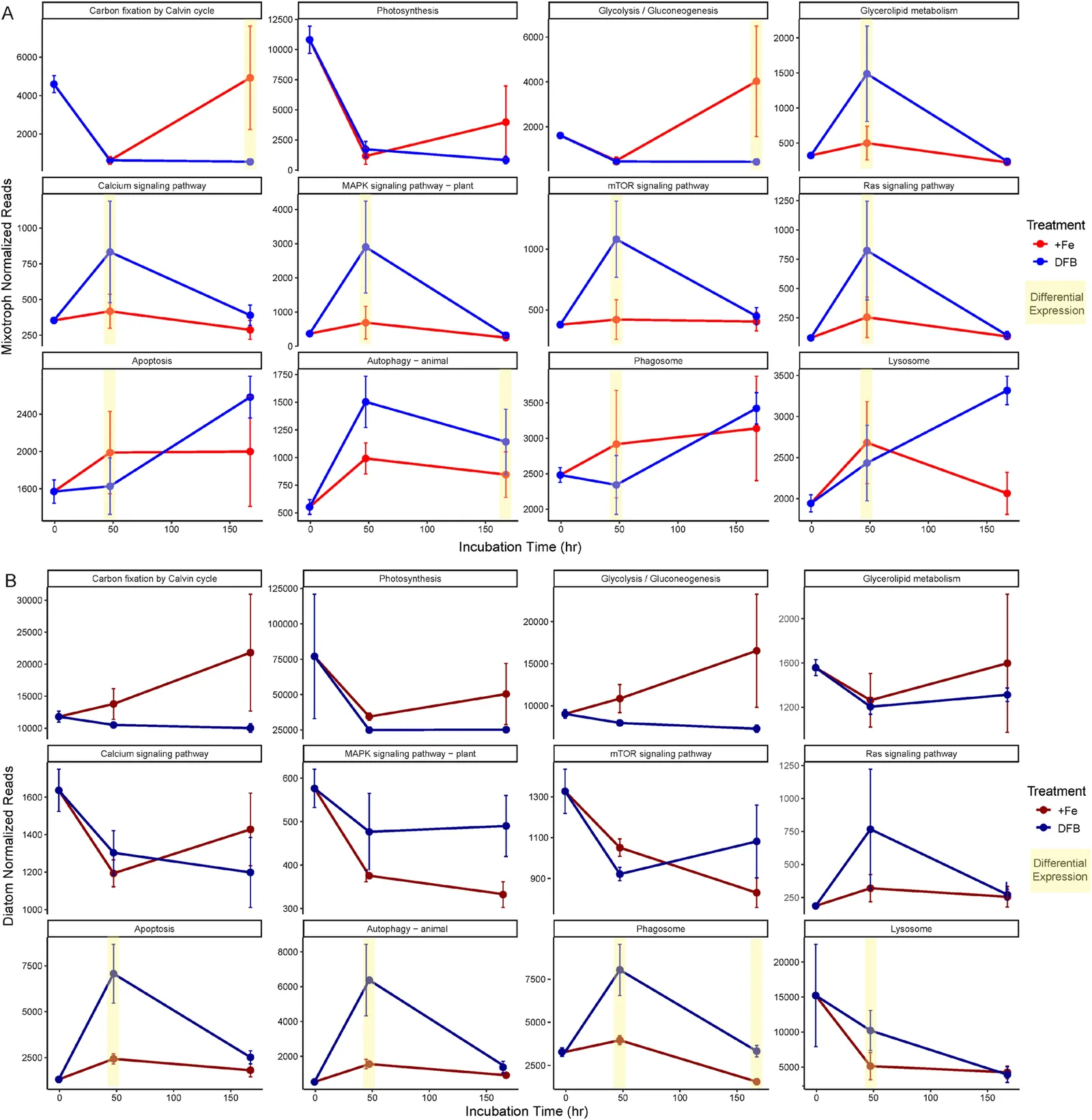
Normalized read expression of KEGG pathways during the iron incubation experiment (mean ± standard deviation) for (A) mixotrophs and (B) diatoms. Iron-addition (+Fe) and desferrioxamine B (DFB) treatments are colored red and blue respectively. Data points correspond to 0 h (T0), 48 h (T1), and 168 h (T2). Differential expression of the pathway between treatments at a designated timepoint is indicated via yellow shading. Pathways related to energy metabolism: Carbon fixation by Calvin Cycle (ko00710), Photosynthesis (ko00195), Glycolysis/Gluconeogenesis (ko00010), & Glycerolipid metabolism (ko00561). Pathways related to signaling: Calcium signaling pathway (ko04020), MAPK signaling pathway—plant (ko04016), mTOR signaling pathway (ko04150), & Ras signaling pathway (ko04014). Pathways related to transport & catabolism/phagotrophy: Apoptosis (ko04210), Autophagy—animal (ko04140), Phagosome (ko04145), & Lysosome (ko04142).

In +Fe diatoms, Calvin cycle and glycolysis pathway counts increased from T1 to T2 but were not significant (Fig. 6B). PSI and LHC expression differed appreciably between mixotrophs and diatoms at T2, with no significant change in expression in PSI subunits and only one differentially expressed LHC subunit in diatoms (Fig. 5). Interestingly, rhodopsin (*Bac_rhodopsin*) was highly expressed in DFB diatoms at T2 but not in mixotrophs (Fig. 3D).

#### Signal transduction

Signal transduction is crucial for several steps of phagotrophy including the recognition and engulfment of prey and phagosome formation [66, 67]. Twenty-two signaling pathways had higher expression in DFB mixotrophs relative to +Fe at T1, including Calcium (ko04020), Mitogen-Activated Protein Kinase (MAPK)—plant (ko04016), mechanistic target of rapamycin (mTOR) (ko04150), and Ras (ko04014) signaling pathways (Fig. 6A). This was likely due to combined contributions from several highly expressed, but not differentially expressed, multi-function protein-encoding genes such as calmodulin (*CALM*), nucleoside-diphosphate kinase (*ndk*), and *RHEB* (Fig. 4). Evidence of phagotrophic signaling in DFB mixotrophs at T1 was also accompanied by three highly expressed protein domains: the EF hand domain (*EF-hand_1*), the Ras family (*Ras*), and the Zn-finger in Ran binding proteins (*zf-RanBP*) (Fig. 3A). In contrast, signal transduction pathways were not significant in DFB diatoms at T1 (Fig. 6B).

#### Cytoskeleton and motility

Cell motility can be used by mixotrophs to help capture prey. Two genes related to the flagella, dynein axonemal assembly factor 1 (*DNAAF1*) and radial spoke head protein 4/6 (*RSPH_4*), were highly expressed in DFB mixotrophs compared to +Fe (Fig. 4). Dynein is considered an indicator of phagotrophy within flagellates [30, 64, 65]. Among other cellular functions, modification of the actin cytoskeleton is important for the engulfment of prey in phagocytosis. Two protein domains related to cytoskeleton were also highly expressed in DFB mixotrophs at T2: *Strumpellin* and *Bystin* (Fig. 3B). The regulation of actin cytoskeleton pathway (ko04810) and its associated genes were not significant in mixotrophs, whereas the pathway and genes related to tubulin, dynein, actin, myosin, and kinesin were highly expressed in DFB diatoms at T2 (Fig. 4).

#### Phagolysosome

After capture, prey is engulfed by the phagosome, which then matures into a phagolysosome, working to digest prey and degrade unwanted materials. The phagosome (ko04145) and lysosome (ko04142) pathways were not differentially expressed in mixotrophs, but distinct trends in expression occurred over time; phagosome expression increased from T1 to T2 while lysosome expression increased continuously from T0 to T2 in DFB mixotrophs (Fig. 6A). V-type ATPases are crucial for acidifying the phagosome and were previously found to be upregulated within mixotrophs and heterotrophs [31, 64]. The overall trend of V-type ATPases for mixotrophs was higher expression in DFB (Fig. 5). Peptidases are responsible for breaking down proteins and common within the phagolysosome; for instance, serine carboxypeptidases were previously upregulated within grazing *Ochromonas* sp. [31]. At T1, zinc carboxypeptidase (*Peptidase_M14*) and serine carboxypeptidase S28 (*Peptidase_S28*) domains were highly expressed in DFB mixotrophs relative to +Fe (Fig. 3A). One lysosomal acid hydrolase, iduronate 2-sulfatase (*IDS*), was highly expressed in DFB mixotrophs at T2 (Fig. 4). Cathepsins are also lysosomal acid hydrolases that have been upregulated within grazing flagellates [30, 65]. Seventeen cathepsins were identified via KEGG, and although none were differentially expressed, six cathepsins had increased expression in DFB mixotrophs at T2 relative to +Fe (Fig. 5).

Apoptosis (ko04210), autophagy—animal (ko04140), phagosome, and lysosome pathways had higher expression in DFB diatoms compared to +Fe at T1 and the phagosome at T2 (Fig. 6B). Apoptosis and phagosome pathways decreased expression from T1 to T2 in DFB diatoms, and lysosome expression decreased consistently from T0 to T2 (Fig. 6B). The differential expression of these pathways in DFB diatoms was likely due to high expression of cathepsin genes, such as cathepsin L (*CTSL*), which is involved in all four pathways mentioned (Fig. 4). Seven other cathepsins were highly expressed in DFB diatoms at T2 (Fig. 5).

## Discussion

### Phytoplankton physiology and composition in response to upwelling

Varying iron status under simulated upwelling conditions impacted the physiology and composition of the phytoplankton communities. Following 48 h into the simulated upwelling event (T1), physiological changes across all treatments were minimal, likely due to low biomass and phytoplankton adjusting to the freshly upwelled environment. Seven days into the simulated upwelling event (T2), growing phytoplankton communities in Ctrl and + Fe treatments resulted in appreciable macronutrient drawdowns, significant increases in biomass, and increased *V*DIC, implying a highly productive community (Fig. 1). The similarities of Ctrl and + Fe treatment responses throughout the experiment were likely due to this site having relatively high dissolved iron concentrations (Table S2) and thus the ambient phytoplankton community was not likely to be iron-limited [68]. There was an apparent abundance of diatoms within these treatments as observed from the increase in large cell chl *a*, drawdown of Si(OH)_4_, high diatom cell counts, and overwhelming proportions of diatom transcripts. This finding is consistent with previous studies where diatoms dominate CCS upwelling communities [5, 6, 18]. Diatoms confer an advantage under high nutrient upwelling conditions by exhibiting a shift-up response through frontloading NO_3_^−^ [69–71]. This shift-up response was shown within our experiment via low *V*NO_3_^−^ in Ctrl and + Fe at T2, implying that these communities were NO_3_^−^ depleted and facing limitations that resulted in declined productivity by Day 11 (T3) (Fig. 1GH). Declined productivity was likely the effect of declined phytoplankton biomass within Ctrl/+Fe by T3, as shown by chl *a* and nanoeukaryote concentrations. While phytoplankton concentrations decreased, bacteria concentrations increased, suggesting possible predation or viral infections for these treatments at T3 (Fig. S3).

Contrary to Ctrl and + Fe, DFB phytoplankton experienced minimal changes in macronutrient drawdown and only small increases in biomass and uptake rates by T2 (Fig. 1). Additionally, calculated POC-to-chlorophyll *a* ratios (although variable between biological replicates) were substantially higher in DFB compared to Ctrl/+Fe at T1 (Table S6). These results suggest that iron functioned as the limiting nutrient in DFB, as anticipated. Iron limitation was previously demonstrated to detriment phytoplankton growth and physiology, specifically within the CCS [4, 18]. The most prolific changes in physiology in DFB eventually occurred at T3, in which macronutrients were drawn down and chl *a* increased. The increased productivity at this timepoint was likely due to chemical dynamics of the iron chelator DFB, resulting in the reduced release of bioavailable iron due to changes in chemical equilibrium, thus allowing for steady phytoplankton growth, albeit at a slower rate [72].

As a result of diatom dominance at T2, RNA taxonomic diversity was minimal in Ctrl and + Fe, resulting in low proportions of reads mapping to mixotrophs. In comparison, DFB exhibited a more diverse taxonomic makeup, as the proportion of mixotroph reads remained above 15% across all DFB timepoints (Fig. 2B). This is consistent with previous findings that nutrient-limited conditions can favor mixotrophs relative to phototrophs and/or heterotrophs [73–75]. It should be noted that different taxa may have different capacities to access siderophore-bound iron, which could influence community composition results in DFB [76, 77]. However, our taxa results still strongly suggest that while iron-replete conditions provided an overwhelming advantage to diatoms, iron-limited conditions curtailed diatom growth, thus allowing more transcriptional activity from mixotrophs and other non-diatom taxa.

Additionally, grazing experiments generally supported higher mixotrophic activity in DFB. Using FLPs to determine mixotrophic grazing has its caveats (using inanimate objects, one prey size, non-ingestion adsorption, etc.) but still provides rates that can be relatively compared among treatments. Although variability was present between replicates, DFB overall supported higher CSGRs, especially compared to +Fe conditions at T1 and T2 (Fig. S6). Additionally, bacteria concentrations in DFB were almost depleted by T3, which could also indicate more grazing pressure via mixotrophs under iron-limited conditions (Fig. S3). Lower grazing rates in +Fe at T1 and T2 were likely influenced by counting high amounts of phototrophic diatom cells, but these values still encapsulate the grazing status of the overall community. At T3, Ctrl/+Fe showed higher grazing rates, but this was most likely due to increased bacteria concentrations that influence the CSGR calculations (Fig. S3).

### Gene expression trends in response to iron status

Key differences in the expression of photosynthetic processes occurred between iron-replete and iron-limited mixotrophs. At T1, expression of these processes was low in both treatments, likely due to changes in light exposure causing photoinhibition [78]. However, by T2, photosynthetic expression (particularly for the Calvin cycle pathway and PSI/LHC subunits) increased in +Fe but stayed low in DFB. One explanation for this trend could be that iron-limited conditions shifted the taxonomic composition of the mixotroph community, such that mixotrophs preferring heterotrophy for energy acquisition were more prevalent. For instance, the chrysophyte genus *Ochromonas*, known for its high grazing rates and primary reliance on heterotrophy for growth [31, 79–81], contributed a higher proportion of reads in DFB compared to +Fe at T2 (Fig. S4). We also believe the reduction of photosynthetic expression in DFB mixotrophs was caused by an intentional change in transcriptional regulation. Some mixotrophs may use phagocytosis to supplement photosynthetic growth under nutrient-limited conditions [73, 75, 82, 83], but this is not the case for all mixotrophs. A previous study showed that although two mixotrophic species were feeding under low-nutrient conditions, their growth was stunted, suggesting that mixotrophy was utilized for survival rather than growth [82]. This finding was corroborated by a recent transcriptomic study of *Pterosperma cristatum*, in which the mixotroph simultaneously upregulated phagotrophy and downregulated photosynthesis under macronutrient limitation [30]. Furthermore, several studies found decreased chl *a* content within mixotrophic species when prey was available, indicating a shift away from photosynthesis [31, 84].

Further evidence of low photosynthetic expression within iron-limited mixotrophs is presented by contrasting the mixotroph response to diatoms. Within diatoms, there were minimal differences in Calvin cycle (Fig. 4) and PSI (Fig. 5) expression between +Fe and DFB at T2. Our results suggest that under iron limitation, strict autotrophic diatoms were sustaining themselves using the typical PSI/Calvin cycle pathway as their primary source of energy. Conversely, expression of PSI/Calvin cycle genes in mixotrophs decreased in DFB, indicating that iron-limited mixotrophs were not heavily utilizing these pathways; instead, they may have shifted to phagotrophy to acquire enough nutrients to sustain themselves. Overall, these findings suggest that the reduced expression of photosynthesis was a reflection of a physiological community shift toward heterotrophic mixotrophs alongside mixotrophs employing an intentional survival strategy to cope with iron limitation.

Another key response that occurred in iron-limited mixotrophs at T1 was their increased expression of signal transduction pathways, which was not observed in diatoms (Fig. 6). Previous studies have found signaling as upregulated within grazing protists, including those under nutrient stress [30, 31, 64, 65, 85]. Our results showed 22 signaling pathways with significantly higher expression in DFB mixotrophs relative to +Fe at T1, as well as several protein domains. High expression of signal transduction may serve as evidence of iron-limited mixotrophs utilizing their phagotrophic capabilities.

We then analyzed the patterns of two pathways involved directly in phagocytosis: the phagosome and the lysosome. Many studies find phagolysosome expression as upregulated within mixotrophs under active grazing [30, 31, 33]. Although no differential expression occurred for these pathways in mixotrophs, their expression trends over time are still biologically relevant, as both pathways experienced increased expression in DFB mixotrophs over time (Fig. 6A). The higher expression of several phagolysosome genes within DFB mixotrophs, such as V-type ATPases and cathepsins, also indicated phagotrophy (Fig. 5).

Diatoms contrasted mixotrophs in that their phagolysosome expression was differentially expressed in DFB at T1 but then decreased from T1 to T2 (Fig. 6B). This might seem to indicate phagotrophy, but no studies have observed phagocytosis within diatoms. Studying the phagolysosome has its difficulties in that it shares genes with several other pathways, including apoptosis and autophagy. Apoptosis and autophagy are employed by stressed cells as a form of self-preservation, such as within diatoms to cope with nutrient stress [86–88]. It is more likely that DFB diatoms were utilizing these pathways rather than the phagolysosome. Cathepsins within diatoms have been associated with programmed cell death and degrading materials within the autophagolysosome, which was likely the role they exhibited in DFB diatoms [89]. Furthermore, cathepsin L is associated with biosilicification within sponges [90]; this gene was differentially expressed in DFB diatoms, and its role within diatoms should be further investigated. Within DFB mixotrophs, autophagy and phagosome pathways do not share similar expression trends over time (Fig. 6A), thus leading us to believe that increased lysosome expression was more influenced by phagolysosome expression. Ultimately, studying phagolysosome expression requires careful interpretation due to the overlap with other metabolic and cellular pathways.

An inconsistency we found was minimal differential expression of actin cytoskeleton and motility genes in iron-limited mixotrophs. Several studies found that genes associated to these processes were upregulated in grazing mixotrophs and heterotrophs [30, 32, 64, 65]. Within our results, most of these genes did not change in expression in mixotrophs. A reason for this discrepancy could be that similar to the phagolysosome, protein-encoding genes involved with capturing and engulfing prey may be important for multiple cellular processes required regardless of iron status. For instance, actin is a highly conserved protein in dinoflagellates that is crucial to general cell structure and development [91, 92]. Interestingly though, diatoms did increase expression of many of these cytoskeleton/motility genes in DFB (Fig. 4). One possible explanation is these genes were utilized for the formation of diatom silica structures, specifically valve morphogenesis [93, 94]. Given that iron limitation can alter silica transport and frustule formation within diatoms, the increased expression of cytoskeleton genes within DFB diatoms is logical [95, 96]. Further research should be conducted to understand the impacts of iron limitation on cell motility and the actin cytoskeleton within both mixotrophs and diatoms.

### Conclusion

Our study provides new insights on the physiological and metatranscriptomic profiles of mixotrophic phytoplankton under various iron states, specifically within upwelling zones. Through our method of defining mixotrophs based on genera, we established a streamlined and reproducible approach to analyze the overall gene expression responses of this large and diverse trophic group. We found that phytoplankton productivity and diatom growth decreased under iron limitation, meaning that future changes in iron bioavailability could have significant impacts on the upwelling ecosystems and the aquaculture industries that rely on them. However, our results suggest that mixotrophs may be able to persist under low dissolved iron concentrations by using phagotrophy for survival. Low iron bioavailability does not necessarily favor the generalist mixotroph strategy; instead, it seems to push mixotrophs to act more like heterotrophs, perhaps using the energy supplied by phagocytosis to temporarily sustain themselves, but not to increase their productivity.

Mixotrophy fundamentally changes the flow of nutrients and energy throughout an ecosystem and, in turn, changes food web dynamics and carbon cycling within the ocean. Mixotrophic behavior could increase trophic transfer that benefits both food chain efficiency and the biological carbon pump relative to strict autotrophic or heterotrophic behavior [20, 25, 26]. Although mixotrophy within an iron-limited upwelling system may not restore the ecosystem to its full potential, its persistence could still make meaningful contributions to the ecosystem that were not recognized previously, thus making mixotrophy worthy of continuous exploration.

## Supplementary Material

Speciale_ISME_Supplementary_Oct2026_Final_rrag100

## Data Availability

Metatranscriptomic raw read data are available in the NCBI online repository and can be accessed under BioProject accession number PRJNA966115. Datasets for physiological measurements, taxonomic composition, and differential expression analyses are publicly available in BCO-DMO under project number 768006 (https://www.bco-dmo.org/project/768006). The metatranscriptomic pipeline code used for this project is publicly available at https://github.com/emilyspeciale/Speciale-Metatranscriptomics.
