# Supplementary material for: Mixotrophy as an alternative strategy under iron-limited upwelling conditions
Source: ISME Commun. 2026 Oct 8;6(1):ycag290. doi: 10.1093/ismeco/ycag290 (PMC13747015; doi:10.1093/ismeco/ycag290)
Supplement: Supplementary file 1 [file speciale_isme_supplementary_oct2026_final_rrag100.pdf]

Supplementary Material

Mixotrophy as an alternative strategy under iron-limited upwelling conditions

Emily V. Speciale, Prisca Lim, Yubeen Jeong, Claire C. Z. Cook, Natalie R. Cohen, Will McClure, Astrid Schnetzer, Claire P. Till, Ralph C. Till, Adrian Marchetti

Corresponding Author: Adrian Marchetti
Email: amarchetti@uvic.ca

This PDF file includes:

- Supplementary Text
- Figures S1 to S6
- Tables S1 to S9
- Supplementary References

Supplementary Text

Detailed methodology for iron incubation experiment

Iron incubation experimental set-up. The seawater for the iron incubation experiment was pumped into a positive pressure trace metal clean laboratory and then into 50-gallon acid-washed high-density polyethylene (HDPE) drums. These drums homogenized the seawater using a Wilden air-operated double diaphragm pump made of polytetrafluoroethylene and acid-washed HDPE tubing. The seawater was then transferred into a total of thirty 20 L low-density polyethylene cubitainers.

Dissolved inorganic macronutrients. Dissolved inorganic macronutrient measurements were collected by filtering 30 mL of seawater through a GF/F filter into an acid-rinsed polypropylene scintillation vial. The filtrate was immediately frozen and then sent to Wetland Biogeochemistry Analytic Services at Louisiana State University and analyzed via their OI Analytical Flow Solutions IV auto analyzer for nitrate + nitrite, nitrite, phosphate (PO_4^{3-}), and silicic acid ($Si(OH)_4$). Detection limits were 0.09 µM for nitrate + nitrite, 0.02 µM for PO_4^{3-}, and 0.02 µM for $Si(OH)_4$. Nitrate (NO_3^-) concentrations were derived by subtracting the concentration of nitrite from nitrate + nitrite. NO_3^- concentrations were also used in calculations for NO_3^- uptake rates.

Dissolved iron. Samples for measurements of dissolved iron (dFe) were collected by filtering 500 mL of seawater directly from cubitainers through acid-cleaned Acropak supor membrane cartridge filters with 0.2 µm pore size. Filtered samples were collected in acid-cleaned LDPE bottles, which were rinsed three times with sample prior to filling. Samples were acidified at sea with optima hydrochloric acid to a pH of ~1.8. DFB samples were then UV irradiated to break DFB molecules and release iron for analysis (UV irradiation is not necessary for separating iron from natural ligands in this method). Samples were analyzed post-cruise using the methods of Lohan et al. [1] with modifications as described in Biller et al. [2]. This method involves preconcentrating iron on a chelating resin; we followed Biller et al. [2] and used Toyopearl Chelate-650 resin, which recovers >93% of Fe(III) at pH 2. Small amounts of optima ammonium hydroxide were added to each sample to bring the pH up from 1.8 to 2 before analysis. Hydrogen peroxide (10 µM) was also added to each sample ten minutes before analysis to quantitatively oxidize Fe(II) to Fe(III) in acidified seawater [3]. After preconcentration, the iron was eluted into a reaction stream with the colorimetric agent N,N-dimethyl-p-phenylenediamine dihydrochloride (DPD), and the signal was quantified with a flow through spectrophotometer. Standard additions were made by spiking low metal seawater with known concentrations of iron, and blanks were assessed by analyzing acidified MilliQ in place of a sample. Reference samples (Geotraces GSC and D1) measured in the same analytical runs as the samples were in line with consensus values.

Size-fractionated chlorophyll *a*. Chlorophyll *a* (chl *a*) measurements were collected by gravity filtering 250 mL of seawater through 5 µm Isopore membrane filters (47 mm) to collect large cells (≥ 5 µm), then vacuum filtering the remaining seawater onto GF/F (25 mm) filters under 100 mmHg of vacuum pressure to collect small cells (< 5 µm). Filters were then rinsed with filtered seawater and stored at -20℃. Chl *a* was extracted on the ship using 90% acetone solution at -20℃ for 24 hours, then measured on a 10-AU fluorometer (Turner Designs, San Jose, CA) using the acidification method [4].

Size-fractionated isotope uptake rates. Isotope uptake rates of dissolved inorganic carbon (DIC) and NO_3^- were assessed by collecting seawater into 1 L polycarbonate bottles (filled to the top, resulting in a total volume of 1.09 L). Each bottle was immediately spiked with $NaH^{13}CO_3$ and $Na^{15}NO_3$, with spiking concentrations varying depending on the ambient DIC and NO_3^- concentrations. The ambient DIC concentrations were estimated at 2000 µM based on literature values [5], while the NO_3^- ambient concentrations were estimated using the Submersible Ultraviolet Nitrate Analyzer (SUNA) [6]. These estimates resulted in adding 200 µM of ^{13}C to

every sample as well as 2, 2, 0.50, and 0.20 µM of ^{15}N at T0, T1, T2, and T3 respectively, to achieve approximate additions of 10% of the ambient DIC and NO_3^- concentrations.

Samples were then placed in the ship incubator for 6 hours, simulated under 25% of incident irradiance. After incubation, the seawater from each bottle was gravity filtered onto 5 µm Isopore membrane filters (47 mm) to collect large cells and vacuum filtered onto pre-combusted (450℃ for 5 hours) GF/F filters to collect small cells. Large cells were then rinsed off the Isopore filter using 0.2 mm filtered seawater and vacuum filtered onto pre-combusted (450℃ for 5 hours) GF/F filters. The standard filtering volume was 1.09 L, but due to accumulation of biomass hindering filtering at the later timepoints, all samples in T2 and T3 had a filtering volume of 0.5 L. Filters were stored in small petri dishes at -20℃ until lab preparation.

To prepare for lab analysis, filters were dried at 60℃ for 24 hours, encapsulated in tin, and pelletized. Pelletized samples were sent to the UC Davis Stable Isotope Facility, where they were analyzed for particulate organic carbon (POC), particulate organic nitrogen (PON), and atom percentages of ^{13}C and ^{15}N using an isotope ratio mass spectrometer (EA-IRMS). POC and PON concentrations (µM) were calculated by dividing the mass of POC/PON (µg) by the respective atomic mass of carbon and nitrogen over the volume filtered. Absolute uptake rates (ρ) of DIC and NO_3^- were calculated using the constant transport model and biomass-normalized uptake rates (V) of DIC and NO_3^- were calculated using the specific uptake model; these calculations utilize actual dissolved NO_3^- concentrations [7].

Flow cytometry. Flow cytometry was performed on a Guava easyCyte HT flow cytometer live on the cruise. The Guava easyCyte HT was outfitted with a blue 488 nm laser. Gain settings were forward scatter (FSC) 4, side scatter (SSC) 1, green fluorescence 2.95, yellow fluorescence 98.7, and red fluorescence 4. Communities of interest were gated based on community recommendations [8]. *Synechococcos* were detected based on high yellow fluorescence and low FSC, picoeukaryotes (~1-3 µm) were detected based on medium yellow and red fluorescence, and nanoeukaryotes (~3-20 µm) were detected based on high red fluorescence. Samples for bacteria abundance were preserved with 0.25% glutaraldehyde and frozen at -80 ºC. Samples were then stained SYBR Green I (Thermo Fisher; 10,000x stock) diluted to a final concentration of 1x and measured on the Guava EasyCyte HT flow cytometer.

FlowCAM analysis. FlowCAM imaging was performed for T2 samples of the iron incubation experiment based on the protocol provided by Pierce et al. 2023 using a Portable Series FlowCam connected to a peristaltic pump [9]. A 10x FlowCell objective was used on autoimage mode for samples preserved in Lugols. The sets of images produced were analyzed using the Yokogawa Fluid Imaging VisualSpreadsheet software. Any images not containing particles (such as bubbles or blurry images) and/or less than 15 µm were deleted from the dataset. For each sample, the first 300 identifiable diatom cells were counted and categorized based on cell morphology and assigned to taxa at the genus level when possible. Final categories included: *Asterionellopsis* spp., *Chaetoceros* spp., *Pseudo-nitzschia* spp., other centric diatoms, and other pennate diatoms. To calculate concentrations of each diatom taxonomy within each sample (cells/L), the following equation was used:

$$\frac{number\ of\ cells\ counted\ \times image\ factor}{volume\ ran\ through\ FlowCAM}$$

The image factor was determined by taking the total number of images for the sample (after quality control) divided the number of images counted for all taxonomy. The volume processed through the FlowCAM was 25 mL for all samples.

FLP experiments for mixotroph grazing. Fluorescently labeled particle (FLP) incubations were performed and analyzed for the iron incubation experiment based on the protocol provided and used by Cook et al. [10]. 500 mL of seawater was pre-screened through 200 µm mesh to remove

mesozooplankton. Fluorescently labeled surrogate microspheres (0.5 μm diameter, Fluoresbrite, Polysciences) were spiked into the volume at concentrations of 10^5 particles mL^{-1}. Immediately after surrogate prey addition, a time zero ($T_{initial}$) sample was taken and preserved. For preservation, 10-30 mL were fixed with 4% glutaraldehyde, incubated at 4 ºC for 30 minutes, filtered onto 3 μm polycarbonate filters, mounted using DAPI Vectashield (Vector Laboratories), and frozen at -20 ºC. Incubations were carried out for one hour in a clear Plexiglass flow-through incubator with in shaded mesh bags emulating *in situ* light availability. After an hour incubation, another sample (T_{final}) was taken and preserved in the same manner as $T_{initial}$.

FLP incubation analysis was performed using an Olympus CKX53 inverted microscope at 400x total magnification. The field-of-view counting method with a calibrated reticle was used. At least 300 chloroplast-containing cells were enumerated on every slide to ensure statistical reliability. Cell specific grazing rate (CSGR, bacteria mixotroph^{-1} hr^{-1}), was calculated using the following equation:

$$CSGR = \frac{\left(\frac{C_{ingested}}{C_{photo}}\right)_{T_{final}} - \left(\frac{C_{ingested}}{C_{photo}}\right)_{T_{initial}}}{t} \times \frac{C_{bacteria}}{C_{FLP}}$$

$C_{ingested}$ is the count of ingested beads divided by the count of all pigmented phototrophic cells (C_{photo}), with $T_{initial}$ values subtracted from T_{final} values to account for non-ingestion adsorption. Values were divided by time of incubation in hours (t) then multiplied by the Bacteria:FLP ratio. $C_{bacteria}$ is the natural concentration of heterotrophic bacteria measured via flow cytometry and C_{FLP} is the concentration of FLPs spiked into the sample (10^5 particles mL^{-1}).

Supplementary Figures

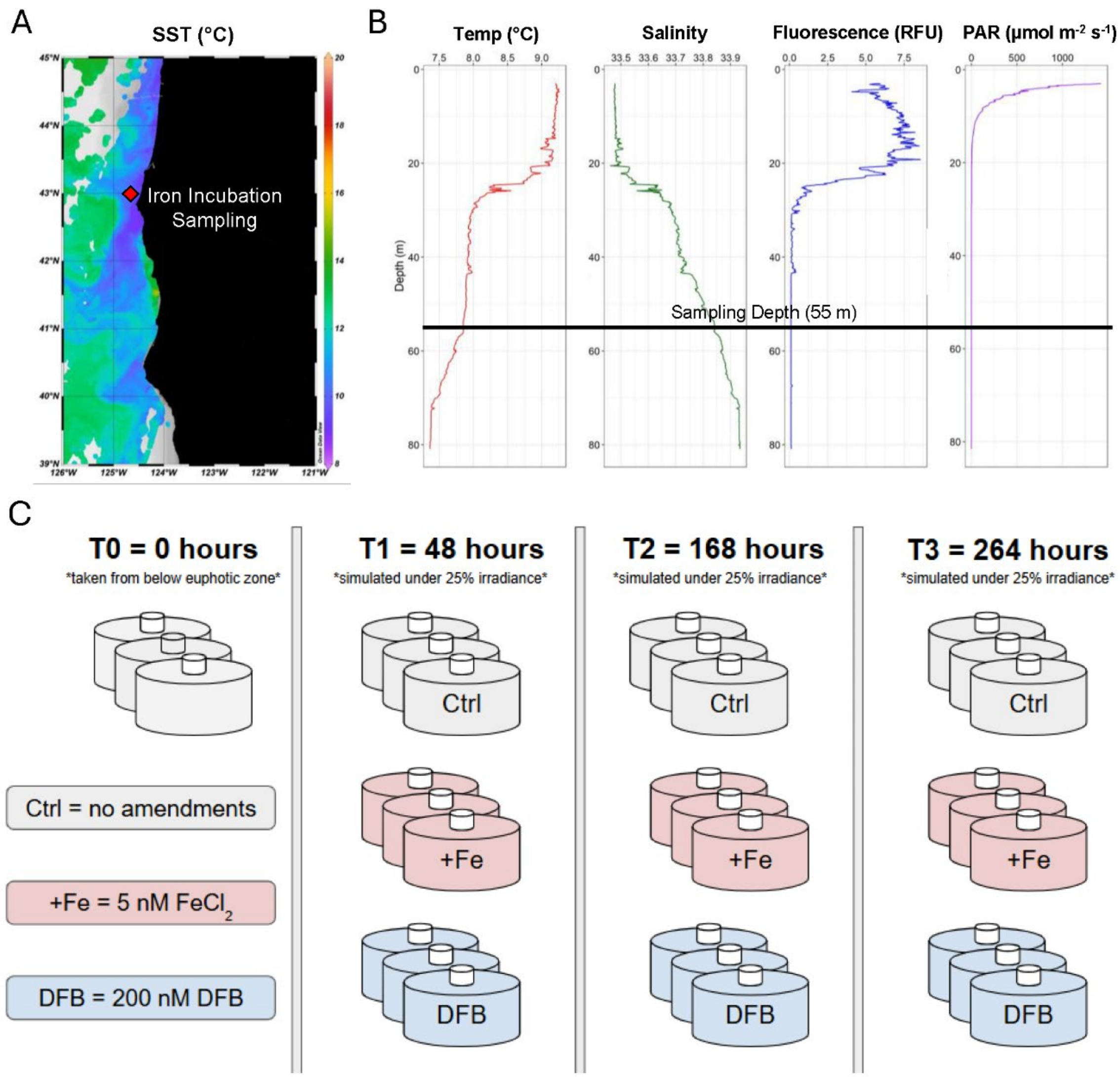

Fig. S1. Iron incubation experiment location and design. **(A)** Approximate location of seawater sampling (43°02'42.7"N, 124°33'07.2"W) on top of remote sensing imagery of sea surface temperature (°C) on May 30th, 2023. **(B)** Depth profiles of temperature (℃), salinity, fluorescence (RFU), and photosynthetically active radiation (PAR) (µmol m^{-2} s^{-1}) at the experiment sampling location, taken from a midday CTD cast on May 30th, 2023. The black line represents the 55 m depth at which seawater was collected. **(C)** Iron incubation experiment design. Three cubitainers were harvested for data collection at the initial timepoint, T0. The remaining cubitainers were assigned to different treatments: nine were unamended (Ctrl), nine were amended with 5 nM $FeCl_2$ (+Fe), and nine were amended with 200 nM desferrioxamine B, a strong iron chelator (DFB). At each subsequent timepoint (T1 = 48 hours, T2 = 168 hours, T3 = 264 hours), three cubitainers from each treatment were harvested for measurements.

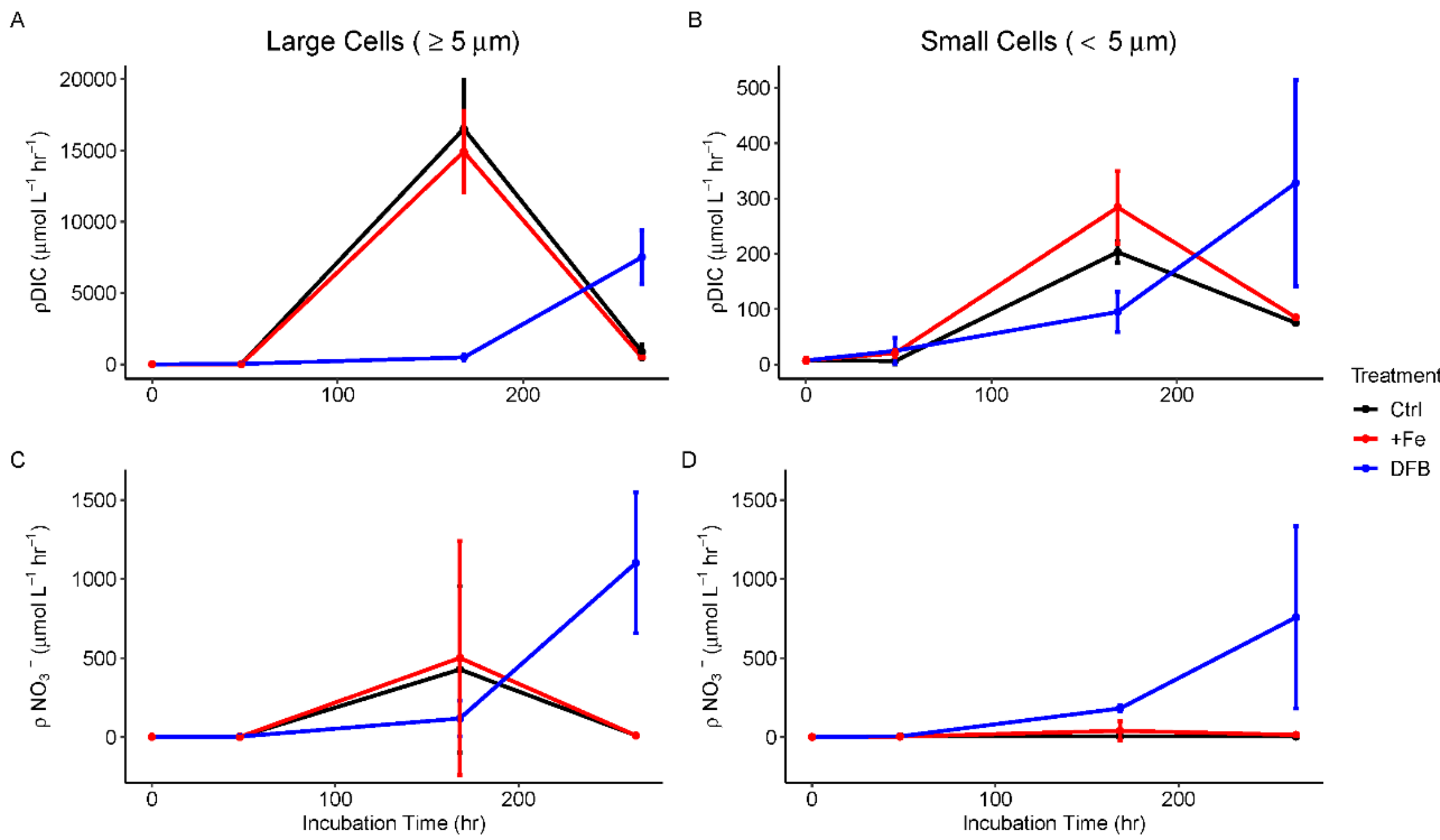

Fig. S2. Absolute uptake rates (mean ± standard deviation) during the iron incubation experiment. Control (Ctrl), iron-addition (+Fe), and desferrioxamine B (DFB) treatments are colored black, red, and blue respectively. Data points correspond to 0 hours (T0), 48 hours (T1), 168 hours (T2), and 264 hours (T3). **(A)** Absolute uptake rates of dissolved inorganic carbon (ρDIC, µmol L^{-1} hr^{-1}) for large cells (≥ 5 µm) and **(B)** small cells (< 5 µm). **(C)** Absolute uptake rates of nitrate (ρNO_3^-, µmol L^{-1} hr^{-1}) for large cells and **(D)** small cells.

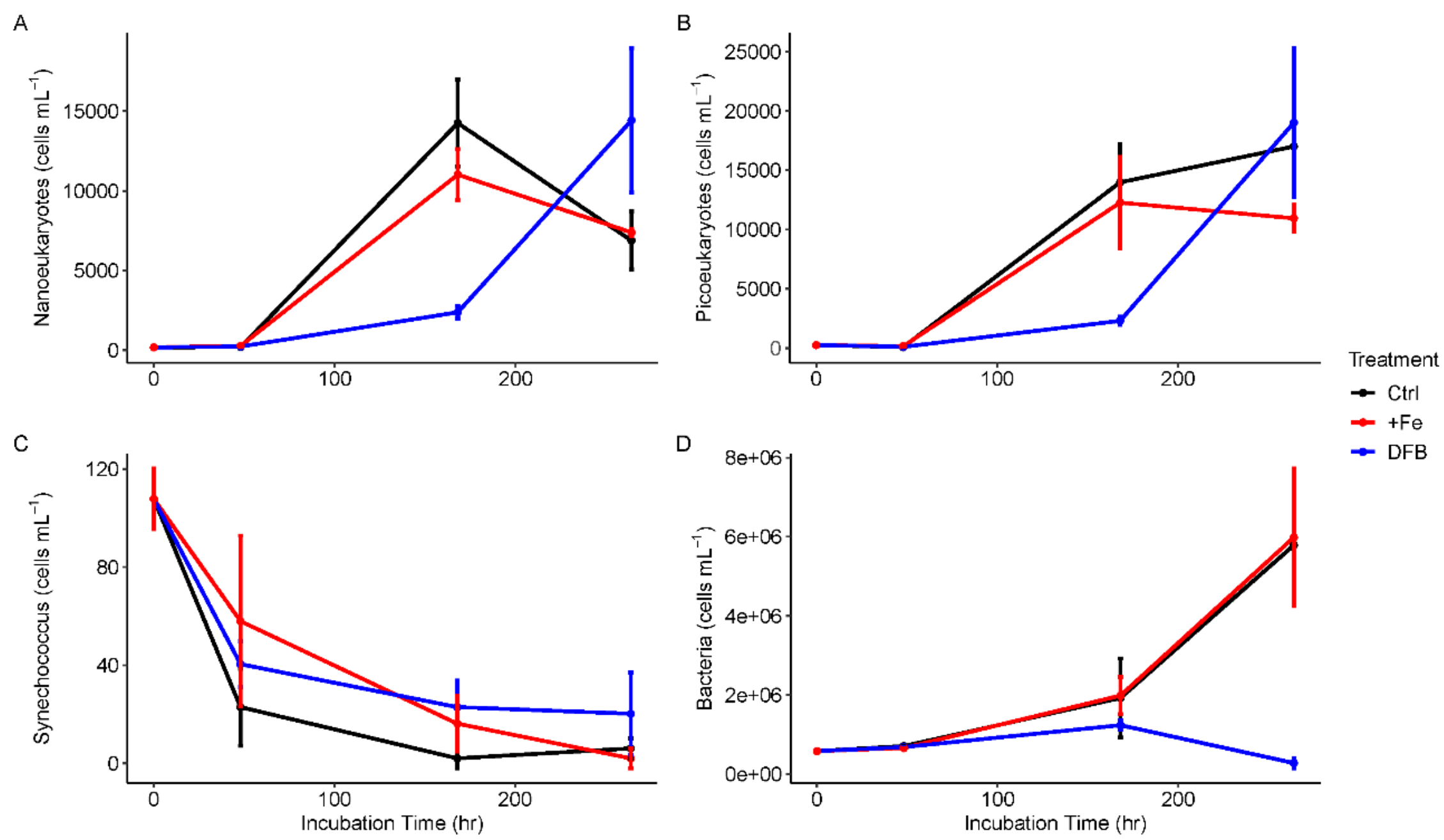

Fig. S3. Concentrations (cells mL^{-1}, mean ± standard deviation) of four taxa groups based on flow cytometry during the iron incubation experiment. Control (Ctrl), iron-addition (+Fe), and desferrioxamine B (DFB) treatments are colored black, red, and blue respectively. Data points correspond to 0 hours (T0), 48 hours (T1), 168 hours (T2), and 264 hours (T3). The four taxa groups measured were **(A)** nanoeukaryotes (~3-20 μm), **(B)** picoeukaryotes (~1-3 μm), **(C)** *Synechococcus* and **(D)** heterotrophic bacteria.

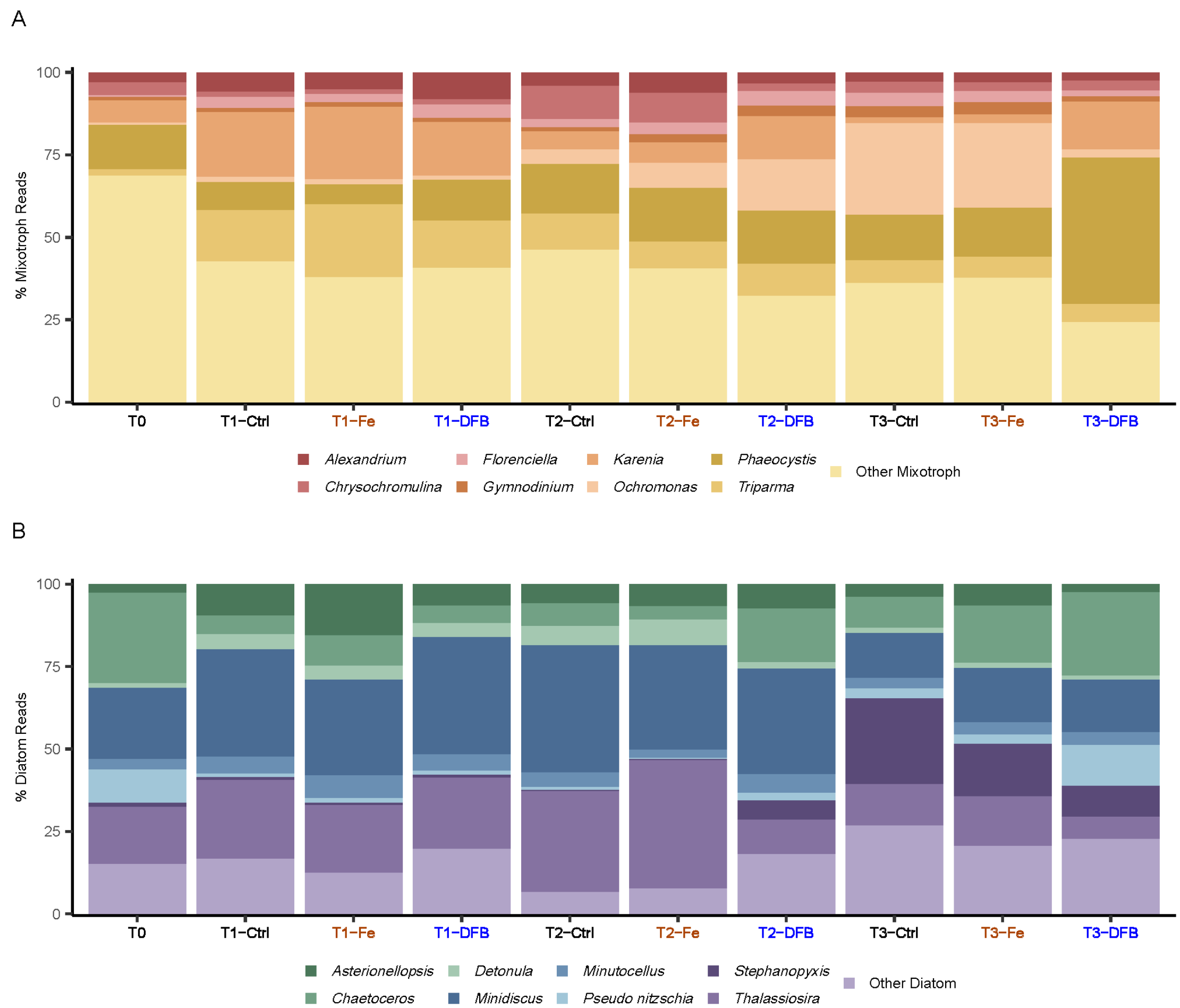

Fig. S4. Mean proportional taxonomic makeup of normalized RNA read counts for each timepoint/treatment within **(A)** only mixotroph reads and **(B)** only diatom reads. Timepoints are 0 hours (T0), 48 hours (T1), 168 hours (T2), and 264 hours (T3) after incubation. Treatments are control (Ctrl), iron-addition (+Fe), and desferrioxamine B addition (DFB). Mixotroph reads were defined by mapping to a constitutive mixotroph genus as defined by the Mixoplankton Database [11]; the eight most abundant mixotroph genera are highlighted, with the rest of the genera grouped into "Other Mixotroph". Diatom reads were defined by mapping to the lineage "Bacillariophyta" as defined by MarFERReT [12]; the eight most abundant diatom genera are highlighted, with the rest of the genera grouped into "Other Diatom".

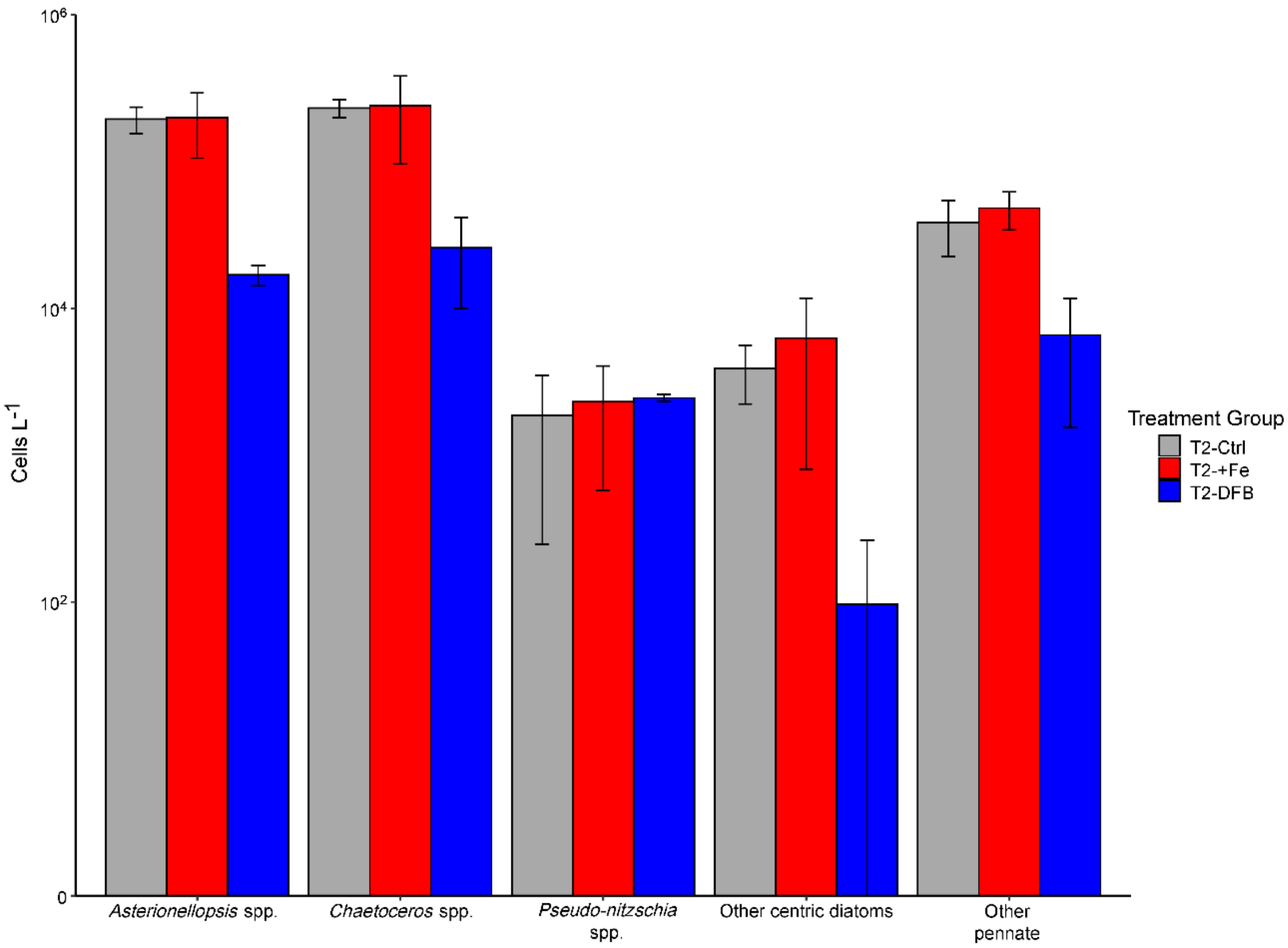

Fig. S5. Concentrations (cells L^{-1}, mean ± standard deviation) of five diatom taxa groups based on FlowCAM imaging at 168 hours (T2) within the incubation experiment. Control (Ctrl), iron-addition (+Fe), and desferrioxamine B (DFB) treatments are colored grey, red, and blue respectively. The diatom taxa groups counted were *Asterionellopsis* spp., *Chaetoceros* spp., *Psuedo-nitzschia* spp., other centric diatoms, and other pennate diatoms.

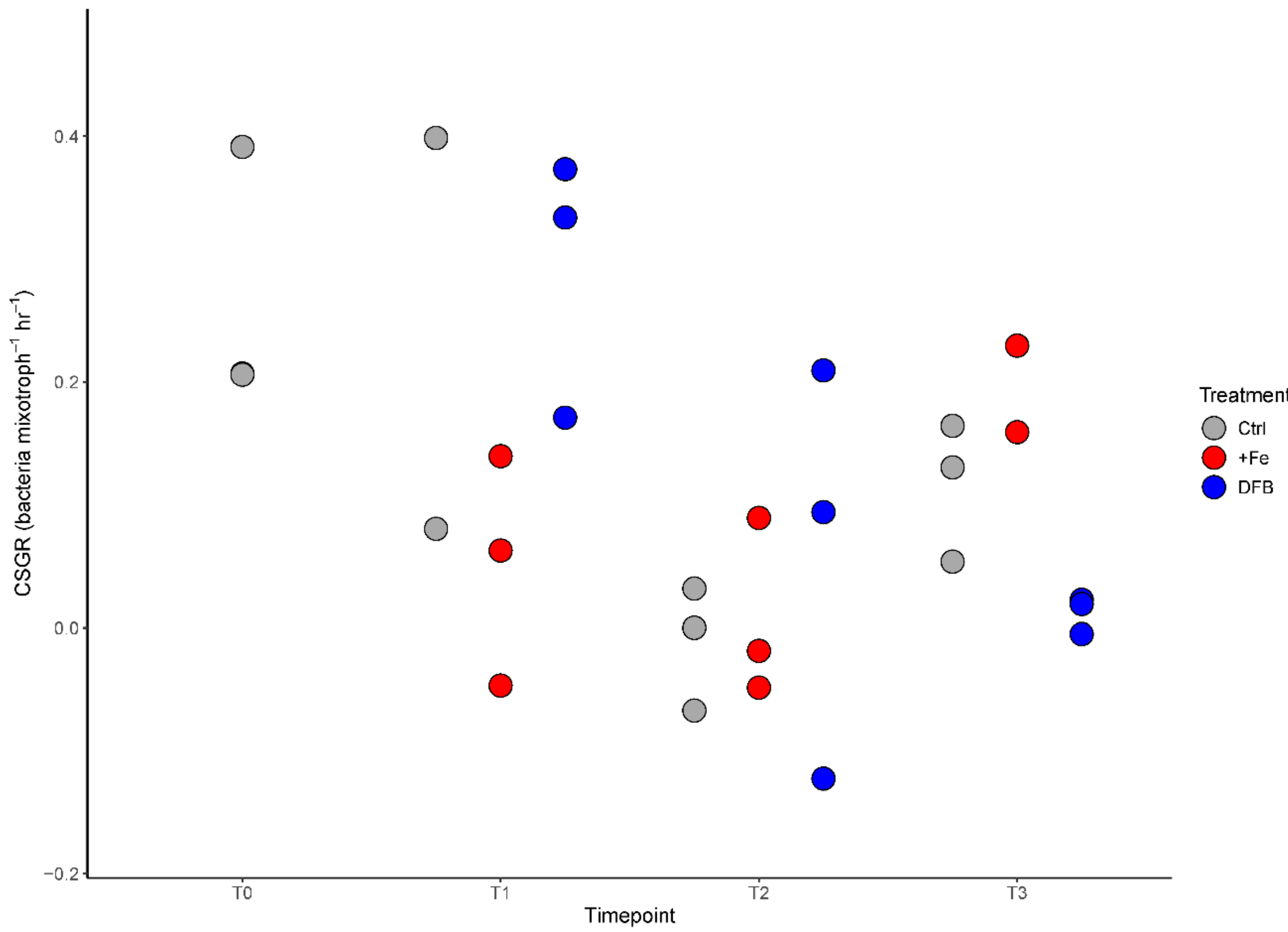

Fig. S6. Cell specific grazing rates (CSGR, bacteria mixotroph^{-1} hr^{-1}) during the iron incubation experiment based on fluorescently labelled particle (FLP) incubations. Control (Ctrl), iron-addition (+Fe), and desferrioxamine B (DFB) treatments are colored grey, red, and blue respectively. Data points correspond to 0 hours (T0), 48 hours (T1), 168 hours (T2), and 264 hours (T3). All replicates are plotted.

Supplementary Tables

Table S1. p-value results from ANOVA tests run for several physiological variables within the incubation experiment. ANOVA tests were run between Control (Ctrl), iron-addition (+Fe), and desferrioxamine B (DFB) treatments at 48 hours (T1), 168 hours (T2), and 264 hours (T3). p-values < 0.05 were deemed statistically significant between treatments.

		p-values		
Measurement Type	Variable	T1	T2	T3
Dissolved nutrients	Nitrate	7.14E-01	3.31E-04	2.12E-04
	Phosphate	7.72E-01	3.37E-04	1.52E-03
	Silicic Acid	8.16E-01	6.37E-03	1.87E-01
Size-fractionated Chl *a*	Large Cells	3.62E-03	1.99E-03	9.68E-01
	Small Cells	3.92E-01	2.11E-01	1.38E-01
Size-fractionated Isotope Uptake Rates	ρNO3- Large	5.55E-01	6.61E-01	2.91E-03
	ρNO3- Small	4.33E-01	2.63E-03	5.12E-02
	*V*NO3- Large	2.50E-01	3.38E-01	1.55E-04
	*V*NO3- Small	3.34E-01	1.58E-03	4.32E-03
	ρDIC Large	5.50E-01	4.85E-04	4.15E-04
	ρDIC Small	3.40E-01	6.00E-03	4.71E-02
	*V*DIC Large	5.79E-01	6.19E-04	2.20E-06
	*V*DIC Small	1.78E-01	3.72E-01	4.06E-03
Flow cytometry	Nanoeukaryotes (cells/mL)	2.24E-02	6.33E-07	5.05E-03
	Picoeukaryotes (cells/mL)	4.54E-02	4.09E-05	5.06E-02
	Synechococcus (cells/mL)	7.58E-02	2.01E-02	7.09E-02
	Bacteria (cells/mL)	2.00E-16	3.00E-01	2.97E-03
FlowCAM	Asterionellopsis (cells/L)	NA	1.42E-02	NA
	Chaetoceros (cells/L)	NA	3.67E-02	NA
	Psuedo-nitzschia (cells/L)	NA	8.60E-01	NA
	Other centrics (cells/L)	NA	1.49E-01	NA
	Other penantes (cells/L)	NA	1.53E-02	NA
FLP incubations	Cell Specific Grazing Rate (bacteria/mixotroph/hr)	1.71E-01	7.21E-01	1.44E-02
RNA Taxonomy (% of Normalized Reads)	Amoebozoa	6.28E-02	3.07E-04	6.57E-01
	Diatoms	2.48E-02	1.23E-04	1.35E-01
	Chlorophyta	3.81E-02	5.61E-03	1.05E-01
	Ciliophora	2.84E-01	1.53E-03	9.97E-01
	Dinophyceae	1.60E-02	2.93E-05	8.78E-01
	Eukaryota	1.87E-01	2.50E-05	4.00E-01
	Haptophyta	1.08E-02	4.64E-03	4.73E-01
	Ochrophyta	9.21E-01	2.08E-04	2.71E-01
	Rhizaria	4.50E-02	4.98E-02	6.01E-01
	All Mixotrophs	5.21E-01	1.50E-04	3.39E-01

Table S2. Mean ± standard deviations of dissolved iron concentrations (nmol/kg) across four timepoints: 0 hours (T0), 48 hours (T1), 168 hours (T2), and 264 hours (T3). Treatment groups were Control (Ctrl), iron-addition (+Fe), and desferrioxamine B (DFB).

Timepoint	Treatment	Dissolved Fe (nmol/kg)
T0	-	6.11 ± 0.12
T1	Ctrl	5.51 ± 0.18
	+Fe	6.77 ± 0.77
	DFB	14.10 ± 0.87
T2	Ctrl	1.28 ± 0.35
	+Fe	1.36 ± 0.71
	DFB	16.8 ± 1.06
T3	Ctrl	1.38 ± 0.36
	+Fe	2.11 ± 0.74
	DFB	17.2 ± 3.59

Table S3. Sequencing statistics for all samples during the iron incubation experiment across four timepoints: 0 hours (T0), 48 hours (T1), 168 hours (T2), and 264 hours (T3). Triplicate samples (A, B, & C) were taken at T0 and for each timepoint/treatment: Control (Ctrl), iron-addition (+Fe), and desferrioxamine B (DFB). Total Reads represents the total number of reads post-trimming. % Mapped is the percentage of total reads mapped to a contig post-alignment. % Taxonomically Annotated, % Pfam Annotated, % KEGG KO Annotated, and % KEGG Pathway Annotated represent the percentage of total reads that annotated to taxonomy (MarFERReT), Pfam function (MarFERReT), KEGG KO ID (eggNOG), and KEGG pathway (eggNOG), respectively **[12–14]**. A summary table for all samples is provided at the bottom, including the total summed value (based on all reads), the average across samples, and the standard deviation.

Timepoint	Treatment	Replicate	Total Reads	% Mapped	% Taxonomically Annotated	% Pfam Annotated	% KEGG KO Annotated	% KEGG Pathway Annotated
T0	-	A	22,481,340	83.1%	39.6%	31.0%	26.9%	22.5%
		B	18,482,670	82.2%	32.9%	25.3%	22.5%	19.0%
		C	26,464,268	83.4%	37.5%	29.7%	24.1%	20.0%
T1	Ctrl	A	27,968,381	87.5%	60.7%	57.3%	50.4%	38.2%
		B	23,913,111	87.1%	59.5%	55.8%	48.9%	36.6%
		C	29,633,965	82.4%	51.1%	47.2%	40.5%	31.0%
	+Fe	A	21,307,220	83.5%	57.0%	52.6%	45.1%	34.7%
		B	23,584,026	72.2%	45.6%	39.8%	32.5%	25.7%
		C	21,300,422	74.0%	43.6%	38.0%	31.6%	24.8%
	DFB	A	28,829,186	87.5%	56.4%	52.5%	46.2%	35.2%
		B	27,361,046	87.0%	58.3%	54.8%	49.1%	37.1%
		C	23,472,984	92.0%	63.7%	60.6%	54.0%	40.8%
T2	Ctrl	A	25,151,963	61.0%	33.8%	27.9%	20.1%	16.4%
		B	20,214,821	57.2%	17.0%	14.6%	12.2%	9.5%
		C	33,216,927	69.4%	28.2%	21.4%	14.3%	11.3%
	+Fe	A	44,142,972	77.9%	36.8%	26.8%	17.7%	13.6%
		B	24,324,275	85.5%	47.4%	35.9%	21.6%	17.7%
		C	21,166,015	70.3%	45.6%	36.3%	23.6%	19.5%
	DFB	A	49,210,396	76.6%	30.4%	25.7%	20.7%	16.7%
		B	35,313,313	73.3%	27.5%	23.5%	19.1%	15.8%
		C	23,408,548	85.6%	45.2%	39.7%	32.7%	26.1%
T3	Ctrl	A	14,592,655	29.7%	15.8%	15.0%	13.4%	11.0%
		B	23,197,162	83.5%	52.8%	45.9%	35.5%	29.0%
		C	21,995,695	74.1%	35.9%	32.2%	29.3%	22.7%
	+Fe	A	25,441,205	56.7%	11.8%	11.2%	11.3%	8.9%
		B	24,832,518	58.6%	24.7%	22.4%	19.9%	16.1%
		C	31,719,459	20.4%	11.6%	11.1%	10.0%	8.1%
	DFB	A	26,845,146	51.8%	21.2%	17.7%	13.8%	11.2%
		B	28,525,240	53.2%	19.9%	17.4%	14.9%	12.3%
		C	29,617,940	79.0%	40.7%	32.7%	24.1%	19.9%
SUM			797,714,869	72.51%	38.01%	32.93%	27.00%	21.29%
AVERAGE			26,590,496	72.19%	38.40%	33.40%	27.53%	21.71%
STANDARD DEVIATION			7,018,806	17.15%	15.37%	14.67%	13.21%	9.77%

Table S4. List of 35 constitutive mixotroph genera (as defined by the Mixoplankton Database) [11] that mapped to metatranscriptomic reads with their associated lineage (as defined by MarFERReT) [12].

Mixotroph Genus	Lineage
Alexandrium	Dinophyceae
Amphidinium	Dinophyceae
Calcidiscus	Haptophyta
Chattonella	Ochrophyta
Chrysochromulina	Haptophyta
Dinobryon	Ochrophyta
Emiliania	Haptophyta
Fibrocapsa	Ochrophyta
Florenciella	Ochrophyta
Gambierdiscus	Dinophyceae
Geminigera	Eukaryota
Gonyaulax	Dinophyceae
Gymnodinium	Dinophyceae
Haptolina	Haptophyta
Heterocapsa	Dinophyceae
Isochrysis	Haptophyta
Karenia	Dinophyceae
Lingulodinium	Dinophyceae
Mantoniella	Chlorophyta
Micromonas	Chlorophyta
Nephroselmis	Chlorophyta
Ochromonas	Ochrophyta
Pelagodinium	Dinophyceae
Phaeocystis	Haptophyta
Prorocentrum	Dinophyceae
Protoceratium	Dinophyceae
Prymnesium	Haptophyta
Pterosperma	Chlorophyta
Pyramimonas	Chlorophyta
Rhizochromulina	Ochrophyta
Scrippsiella	Dinophyceae
Symbiodinium	Dinophyceae
Synchroma	Ochrophyta
Triparma	Ochrophyta
Tripos	Dinophyceae

Table S5. Differential expression results of iron-addition (+Fe) vs. desferrioxamine B (DFB) treatments for mixotrophs and diatoms at 48 hours (T1) and 168 hours (T2). Differential expression analyses were conducted for Pfam protein domains, KEGG KO IDs (KEGG genes), and KEGG pathways. The number (#) of domains/genes/pathways were calculated by grouping reads based on their unique ID prior to differential expression analysis. A domain/gene/pathway was considered to have higher expression in a treatment if the magnitude of log2FoldChange (L2FC) > 1, and a domain/gene/pathway was considered differentially expressed (DE) in a treatment if the p-adjusted value (p-adj) was < 0.05 and |L2FC| > 1.

		Mixotrophs		Diatoms	
		T1	T2	T1	T2
Pfam	# Domains	4,862	4,862	4,453	4,453
	% Higher Expression in +Fe	17.0%	25.4%	9.8%	24.8%
	% +Fe DE	0.0%	3.4%	0.1%	3.6%
	% Higher Expression in DFB	14.9%	26.4%	17.6%	26.1%
	% DFB DE	0.4%	0.5%	0.5%	5.8%
KEGG Genes	# Genes	8,103	8,103	6,311	6,311
	% Higher Expression in +Fe	18.0%	23.4%	9.1%	23.5%
	% +Fe DE	0.0%	2.3%	0.0%	3.2%
	% Higher Expression in DFB	13.9%	24.7%	19.7%	31.9%
	% DFB DE	0.0%	0.3%	0.2%	7.5%
KEGG Pathways	# Pathways	431	431	424	424
	% Higher Expression in +Fe	3.9%	12.8%	2.1%	9.4%
	% +Fe DE	0.0%	5.1%	0.0%	1.9%
	% Higher Expression in DFB	18.1%	4.9%	10.1%	14.6%
	% DFB DE	15.5%	0.5%	3.1%	8.0%

Table S6. Mean ± standard deviations of Particulate Organic Carbon (POC) to chlorophyll *a* ratios for large (≥ 5 μm) and small (< 5 μm) cells across four timepoints: 0 hours (T0), 48 hours (T1), 168 hours (T2), and 264 hours (T3). Treatment groups were Control (Ctrl), iron-addition (+Fe), and desferrioxamine B (DFB).

		POC:Chlorophyll *a* Ratio	
Timepoint	Treatment	Large Cells (≥ 5 μm)	Small Cells (< 5 μm)
T0	-	421.68 ± 155.72	869.68 ± 413.74
T1	Ctrl	406.51 ± 270.19	1672.19 ± 1993.72
	+Fe	138.12 ± 104.18	2548.85 ± 1426.17
	DFB	6227.59 ± 9081.1	18630.71 ± 22239.59
T2	Ctrl	62.58 ± 6.77	238.31 ± 113.97
	+Fe	42.77 ± 8.51	343.72 ± 90.98
	DFB	78.47 ± 41.83	288.93 ± 76.46
T3	Ctrl	265.95 ± 256.48	2009.86 ± 1196.67
	+Fe	154.60 ± 38.29	1712.48 ± 626.19
	DFB	207.16 ± 92.32	130.94 ± 56.14

Table S7. Glossary of Pfam terms for Figure 3. The Pfam short name, which is shown in the figure, is accompanied by its number ID (Pfam ID) and description (Pfam Long Name).

Pfam Short Name	Pfam ID	Pfam Long Name
Aa_trans	PF01490	Transmembrane amino acid transporter protein
Actin	PF00022	Actin
Bac_rhodopsin	PF01036	Bacteriorhodopsin-like protein
Bystin	PF05291	Bystin
CA_like	PF10563	Delta carbonic anhydrase
Calreticulin	PF00262	Calreticulin family
CaMKII_AD	PF08332	Calcium/calmodulin dependent protein kinase II association domain
Copper-bind	PF00127	Copper binding proteins, plastocyanin/azurin family
CS	PF04969	CS domain
Cytochrom_B_C	PF00032	Cytochrome b(C-terminal)/b6/petD
Cytochrome_CBB3	PF13442	Cytochrome C oxidase, cbb3-type, subunit III
DUF305	PF03713	Domain of unknown function (DUF305)
EF-hand_1	PF00036	EF hand
F_bP_aldolase	PF01116	Fructose-bisphosphate aldolase class-II
FA_desaturase	PF00487	Fatty acid desaturase
FAD_binding_3	PF01494	FAD binding domain
Fea1	PF07692	Low iron-inducible periplasmic protein
Ferric_reduct	PF01794	Ferric reductase like transmembrane component
Flavodoxin_1	PF00258	Flavodoxin
Frag1	PF10277	Frag1/DRAM/Sfk1 family
FTR1	PF03239	Iron permease FTR1 family
Glyco_transf_34	PF05637	galactosyl transferase GMA12/MNN10 family
Glyco_transf_8	PF01501	Glycosyl transferase family 8
Glycolytic	PF00274	Fructose-bisphosphate aldolase class-I
Gp_dh_C	PF02800	Glyceraldehyde 3-phosphate dehydrogenase, C-terminal domain
H_PPase	PF03030	Inorganic H+ pyrophosphatase
His_Phos_1	PF00300	Histidine phosphatase superfamily (branch 1)
HMG_box	PF00505	HMG (high mobility group) box
KH_1	PF00013	KH domain
Lipase_3	PF01764	Lipase (class 3)
LUC7	PF03194	LUC7 N_terminus
Methyltransf_23	PF13489	Methyltransferase domain
NAR2	PF16974	High-affinity nitrate transporter accessory, Ig-like domain
NadA	PF02445	Quinolinate synthetase A protein
NDK	PF00334	Nucleoside diphosphate kinase
NIR_SIR	PF01077	Nitrite and sulphite reductase 4Fe-4S domain
ParcG	PF10274	Parkin co-regulated protein
PARG_cat	PF05028	Poly (ADP-ribose) glycohydrolase (PARG), Macro domain fold
Peptidase_C1	PF00112	Papain family cysteine protease
Peptidase_M14	PF00246	Zinc carboxypeptidase
Peptidase_M24	PF00557	Metallopeptidase family M24
Peptidase_M3	PF01432	Peptidase family M3
Peptidase_S10	PF00450	Serine carboxypeptidase
Peptidase_S28	PF05577	Serine carboxypeptidase S28
peroxidase	PF00141	Peroxidase
PetN	PF03742	PetN
PGK	PF00162	Phosphoglycerate kinase
PHO4	PF01384	Phosphate transporter family
PsaL	PF02605	Photosystem I reaction centre subunit XI
PSI_PSAK	PF01241	Photosystem I psaG / psaK
Ras	PF00071	Ras family
Rieske	PF00355	Rieske [2Fe-2S] domain
RuBisCO_large	PF00016	Ribulose bisphosphate carboxylase large chain, catalytic domain
RuBisCO_small	PF00101	Ribulose bisphosphate carboxylase, small chain
Sec7	PF01369	Sec7 domain
SIS	PF01380	SIS domain
Strumpellin	PF10266	Hereditary spastic paraplegia protein strumpellin
Sulfotransfer_1	PF00685	Sulfotransferase domain
Sulfotransfer_3	PF13469	Sulfotransferase family
Thioredoxin_8	PF13905	Thioredoxin-like
Tubulin	PF00091	Tubulin/FtsZ family, GTPase domain
U-box	PF04564	U-box domain
vATP-synt_E	PF01991	ATP synthase (E/31 kDa) subunit
zf-RanBP	PF00641	Zn-finger in Ran binding protein and others
zf-RING_2	PF13639	Ring finger domain

Table S8. Glossary of KEGG genes for Figure 4. The KEGG gene name, which is shown in the figure, is accompanied by its number ID (KEGG ko) and description.

KEGG Name	KEGG ko	Description
fldA	K03839	flavodoxin I
petF	K02639	ferredoxin
nirA	K00366	ferredoxin-nitrite reductase
NR	K10534	nitrate reductase (NAD(P)H)
NRT2	K02575	MFS transporter, NNP family, nitrate/nitrite transporter
AACS	K01907	acetoacetyl-CoA synthetase
ALDO	K01623	fructose-bisphosphate aldolase, class I
FBA	K01624	fructose-bisphosphate aldolase, class II
GAPDH	K00134	glyceraldehyde 3-phosphate dehydrogenase (phosphorylating)
FRD	K18561	FAD-dependent fumarate reductase
fumC	K01679	fumarate hydratase, class II
mdh	K00024	malate dehydrogenase
rbcL	K01601	ribulose-bisphosphate carboxylase large chain
rbcS	K01602	ribulose-bisphosphate carboxylase small chain
CYC	K08738	cytochrome c
nadA	K03517	quinolinate synthase
NDUFA5	K03949	NADH dehydrogenase (ubiquinone) 1 alpha subcomplex subunit 5
NDUFB9	K03965	NADH dehydrogenase (ubiquinone) 1 beta subcomplex subunit 9
NQO1	K00355	NAD(P)H dehydrogenase (quinone)
POR	K00327	NADPH-ferrihemoprotein reductase
CALM	K02183	calmodulin
CDC42	K04393	cell division control protein 42
ndk	K00940	nucleoside-diphosphate kinase
RAB5C	K07889	Ras-related protein Rab-5C
RAB7L1	K07916	Ras-related protein Rab-7L1
RHEB	K07208	Ras homolog enriched in brain
ANKRD1	K21433	ankyrin repeat domain-containing protein 1
CAPZA	K10364	F-actin-capping protein subunit alpha
CAPZB	K10365	F-actin-capping protein subunit beta
DNAAF1	K19750	dynein axonemal assembly factor 1
KIF1	K10392	kinesin family member 1
MYLK	K00907	myosin-light-chain kinase
TUBA	K07374	tubulin alpha
TUBB	K07375	tubulin beta
RSPH4_6	K19756	radial spoke head protein 4/6
CTSL	K01365	cathepsin L
IDS	K01136	iduronate 2-sulfatase
PRCP	K01285	lysosomal Pro-X carboxypeptidase
glmS	K00820	glutamine---fructose-6-phosphate transaminase (isomerizing)
MIB	K10645	E3 ubiquitin-protein ligase mind-bomb
paaG	K15866	2-(1,2-epoxy-1,2-dihydrophenyl)acetyl-CoA isomerase
WDR35	K19674	WD repeat-containing protein 35
GAR1	K11128	H/ACA ribonucleoprotein complex subunit 1
MRT4	K14815	mRNA turnover protein 4
phr	K01669	deoxyribodipyrimidine photo-lyase
TOP2	K03164	DNA topoisomerase II

Table S9. KEGG genes (symbols listed) included in and plotted for each KEGG Gene Group in Fig. 6.

KEGG Gene Group	KEGG Genes Names
PSI	psaA; psaB; psaC; psaD; psaE; psaF; psaH; psaI; psaK; psaL; psaN; psaG; psaO
PSII	psbA; psbB; psbC; psbD; psbF; psbL; psbM; psbO; psbP; psbU; psbV; psb W; psbY; psbR; psbS; psbQ; psb27; psb28
Cytochrome b6f	petA; petC; petD; petE; petF; petH; petN; petJ
LHC	LHCA1; LHCA2; LHCA3; LHCA4; LHCA5; LHCB1; LHCB2; LHCB3; LHCB4; LHCB5; LHCB6; LHCB7
F-type ATPase	ATPF0A; ATPF0B; ATPF0C; ATPF1A; ATPF1B; ATPF1D; ATPF1E; ATPF1 G; ATPeF0A; ATPeF0B; ATPeF0C; ATPeF0F; ATPeF1A; ATPeF1B; ATPeF 1D; ATPeF1E; ATPeF1G; ATPeF0O; ATPeF0D; ATPeFG
V-type ATPase	ATPeV1H; ATPeV1A; ATPeV0D; ATPeV1B; ATPeV1C; ATPeV1D; ATPeV1 E; ATPeV1F; ATPeV1G; ATPeV0E; ATPeV0A; ATPeV0C; ATPeV0B; ATPe VS1
Cathepsins	CTSC; CTSG; CTSB; CTSL; CTSH; CTSS; CTSK; CTSF; CTSO; CTSL2; C TSD; CTSE; CTSZ; CTSW; CTSP; CTSM; CTSR; CTSA

Supplementary References

1. Lohan MC, Aguilar-Islas AM, Bruland KW. Direct determination of iron in acidified (pH 1.7) seawater samples by flow injection analysis with catalytic spectrophotometric detection: Application and intercomparison: Direct determination of iron in seawater at pH 1.7. *Limnol Oceanogr Methods* 2006;**4**(6):164–71. https://doi.org/10.4319/lom.2006.4.164.

2. Biller DV, Coale TH, Till RC, Smith GJ, Bruland KW. Coastal iron and nitrate distributions during the spring and summer upwelling season in the central California Current upwelling regime. *Continental Shelf Research* 2013;**66**:58–72. https://doi.org/10.1016/j.csr.2013.07.003.

3. Lohan MC, Aguilar-Islas AM, Franks RP, Bruland KW. Determination of iron and copper in seawater at pH 1.7 with a new commercially available chelating resin, NTA Superflow. *Analytica Chimica Acta* 2005;**530**(1):121–9. https://doi.org/10.1016/j.aca.2004.09.005.

4. Parsons TR, Maita Y, Lalli CM. *A Manual of Chemical and Biological Methods for Seawater Analysis*. repr, Oxford: Pergamon Press, 1985.

5. Fassbender AJ, Sabine CL, Feely RA, Langdon C, Mordy CW. Inorganic carbon dynamics during northern California coastal upwelling. *Continental Shelf Research* 2011;**31**(11):1180–92. https://doi.org/10.1016/j.csr.2011.04.006.

6. Johnson KS, Coletti LJ. In situ ultraviolet spectrophotometry for high resolution and long-term monitoring of nitrate, bromide and bisulfide in the ocean. *Deep Sea Research Part I: Oceanographic Research Papers* 2002;**49**(7):1291–305. https://doi.org/10.1016/S0967-0637(02)00020-1.

7. Dugdale RC, Wilkerson FP. The use of ^{15} N to measure nitrogen uptake in eutrophic oceans; experimental considerations1,2. *Limnology & Oceanography* 1986;**31**(4):673–89. https://doi.org/10.4319/lo.1986.31.4.0673.

8. Thyssen M, Grégori G, Créach V, Lahbib S, Dugenne M, Aardema HM *et al.* Interoperable vocabulary for marine microbial flow cytometry. *Front Mar Sci* 2022;**9**:975877. https://doi.org/10.3389/fmars.2022.975877.

9. Pierce E, Torano O, Lin Y, Schnetzer A, Marchetti A. Comparison of advanced methodologies for diatom identification within dynamic coastal communities. *Limnology & Ocean Methods* 2023;**21**(11):687–702. https://doi.org/10.1002/lom3.10575.

10. Cook CCZ, Ewton EM, Marchetti A, Menden-Deuer S, Millette NC, Slomka S *et al.* Evaluating acidotropic dyes for detecting mixotrophy in protists: Insights from cultures and field communities. Preprint, Ecology, 1 Oct. 2025. https://doi.org/10.1101/2025.09.29.679303.

11. Mitra A, Caron DA, Faure E, Flynn KJ, Leles SG, Hansen PJ *et al.* The Mixoplankton Database (MDB): Diversity of photo-phago-trophic plankton in form, function, and distribution across the global ocean. *J Eukaryotic Microbiology* 2023;**70**(4):e12972. https://doi.org/10.1111/jeu.12972.

12. Groussman RD, Blaskowski S, Coesel SN, Armbrust EV. MarFERReT, an open-source, version-controlled reference library of marine microbial eukaryote functional genes. *Sci Data* 2023;**10**(1):926. https://doi.org/10.1038/s41597-023-02842-4.

13. Cantalapiedra CP, Hernández-Plaza A, Letunic I, Bork P, Huerta-Cepas J. eggNOG-mapper v2: Functional Annotation, Orthology Assignments, and Domain Prediction at the

Metagenomic Scale. *Molecular Biology and Evolution* 2021;**38**(12):5825–9. https://doi.org/10.1093/molbev/msab293.

14. Kanehisa M. KEGG: Kyoto Encyclopedia of Genes and Genomes. *Nucleic Acids Research* 2000;**28**(1):27–30. https://doi.org/10.1093/nar/28.1.27.
